# Laser-Processed 2D Germanane on Graphene for Organohydrogel-Based Zinc-Ion Hybrid Capacitors

**DOI:** 10.1021/acsnano.5c13803

**Published:** 2026-02-27

**Authors:** Sujit Deshmukh, Keval K. Sonigara, Rostislav Langer, Michal Otyepka, Martin Pumera

**Affiliations:** † Future Energy and Innovation Laboratory, Central European Institute of Technology, 48274 Brno University of Technology, Purkyňova 123, Brno 61200, Czech Republic; ‡ 48207 IT4Innovations, VSB − Technical University of Ostrava, 17. listopadu 2172/15, Ostrava-Poruba 708 00, Czech Republic; § Regional Centre of Advanced Technologies and Materials, Czech Advanced Technology and Research Institute (CATRIN), Palacký University Olomouc, Olomouc 779 00, Czech Republic; ∥ Faculty of Electrical Engineering and Computer Science, VSB - Technical University of Ostrava, 17. listopadu 2172/15, Ostrava 70800, Czech Republic; ⊥ Department of Medical Research, China Medical University Hospital, China Medical University, No. 91 Hsueh-Shih Road, Taichung 40402, Taiwan; # Department of Chemical and Biomolecular Engineering, Yonsei University, 50 Yonsei-ro, Seodaemun-gu, Seoul 03722, Korea

**Keywords:** Germanane, Organohydrogel, Graphene, Laser, Zn-ion hybrid capacitor, Quasi-solid-state electrolyte

## Abstract

Group 14 monoelemental two-dimensional (2D) materials beyond graphene, such as silicene and germanene, have gained significant attention in the scientific community. Covalent functionalization of germanene with hydrogen and methyl leads to germanane (hydrogen/methyl-terminated germanene; HGe/MGe). While the optical and electronic properties of HGe and MGe were explored previously, there is no report on their zinc ion storage electrochemistry. Though the layered HGe/MGe sheets have tunable interlayer spacing, which cushions the volume expansion during ion storage, their inferior electrical conductivity limits the charge transfer kinetics. Herein, we demonstrate a single-step, facile approach for in situ decoration of 2D HGe/MGe sheets over laser-induced graphene (LIG) using a pulsed laser and examine their morphological, chemical, and electrochemical (EC) characteristics. The HGe/MGe-decorated LIG is tested as a cathode for a zinc ion hybrid capacitor (ZHC) in an aqueous electrolyte and polyacrylamide organohydrogel to unveil the selective sites for zinc ion electrochemistry by experimental and theoretical aspects. This ZHC design enables a notable Zn^2+^ storage capacity (104 F g^–1^ @ 0.25 A g^–1^ in aqueous electrolyte) for HGe-decorated LIG, whereas MGe-decorated LIG records impressive cyclic stability (capacity retention 83% after 12000 cycles). Density functional theory calculations elucidate favorable adsorption of Zn at MGe and HGe networks. These findings summarize the applicability of 2D functionalized germanane, which has the potential to expand by numerous alkyl chains and terminal groups for targeted energy storage applications.

## Graphical abstract

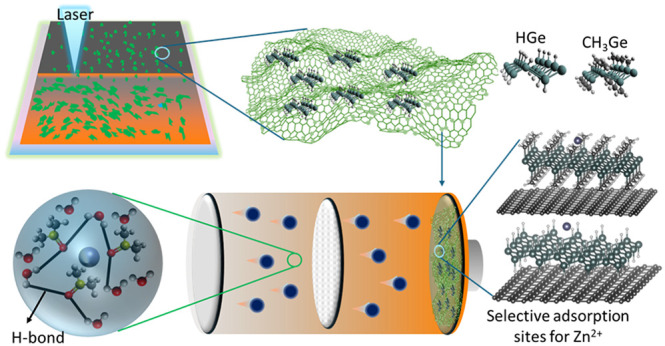

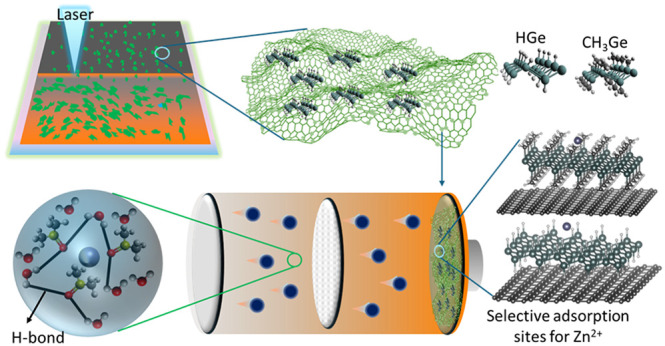

## Introduction

Metal-ion hybrid capacitors, which integrate an anode (battery-type) with a cathode (capacitive-type), have garnered significant attention due to their high capacity and power output coupled with excellent long-term stability.^1–3^ Among these, aqueous ZHCs are particularly notable for their safety, cost-effectiveness, and environmental compatibility, especially in the cathode design module.^4^ Particularly, the development of carbon cathodes within ZHCs has seen remarkable progress, with materials such as N-doped hierarchically porous carbon,^5^ carbon hollow spheres,^6^ asymmetric hollow bowl-like carbon structures,^7^ and two-dimensional carbon materials showing good promise.^8,9^ These carbon cathodes offer high power output and long cyclic stability due to the reversible adsorption/desorption of Zn^2+^ ions. However, commercial porous carbons often suffer from inadequate Zn^2+^ storage capacity due to inadequate sites for favorable adsorption/desorption of Zn^2+^.^8^ Therefore, it necessitates advanced engineering of carbonaceous materials to increase the density of Zn^2+^ adsorption sites.

Beyond two-dimensional (2D) carbon, a novel class of 2D monolayers of group 14 elements, silicene and germanene, is attracting considerable scientific interest.^10,11^ Unlike graphene, which consists of sp^2^-hybridized planar carbon layer, these group 14 analogues feature sp^2^–sp^3^-hybridized systems with folded honeycomb arrangements.^10,11^ Germanium (Ge), silicon (Si), and their 2D forms, i.e., germanene and silicene, exhibit tunable physical and chemical properties through covalent functionalization, effectively converting them into 2D Xanes (e.g., from germanene to germanane) by termination with hydrogen atoms, methyl, propyl, or other alkyl groups.^12–15^ This versatility opens new avenues for the development of advanced semiconductor applications.

Particularly, Ge demonstrates a high specific capacity, an electrical conductivity of 2.1 S m^–1^ (∼10^4^ times higher than silicon), and rapid lithium-ion diffusivity (10^–12^ to 10^–8^ cm^2^ s^–1^), making it a promising material for lithium-ion batteries with outstanding life cycle and excellent rate performance.^16,17^ Its distinctive physical and chemical properties, modulated by tunable layers (few-layer to bulk) and surface functional groups attachment, e.g., germanane (Ge_6_H_6_) or methyl germanane (Ge_6_(CH_3_)_6_), also make it appropriate for various applications, including electronics,^18^ optics,^13^ transistors,^19^ and energy storage systems.^20^ However, a significant challenge for Ge in energy storage is its substantial volume expansion (about 300%) during the lithium-alloying process. This expansion can lead to the fracturing of Ge particles and delamination between the active material and the current collector, causing fast capacity degradation during long-term cycling.^21–23^ To mitigate these issues, researchers have pursued several strategies: nanocrystallization into various forms, such as nanowires,^24^ nanotubes,^25^ and nano-Ge films,^26^ or creating a heterostructure with carbon compounds, such as amorphous carbon^27^ to provide effective matrix buffering. These approaches aim to accommodate volume adjustments during alloying/dealloying reactions, enhancing the durability and performance of Ge-based materials in energy storage applications.

2D heterostructures offer unique opportunities by stacking various 2D materials such as MXenes, boron nitrides, transition metal dichalcogenides (TMDs), graphene, and Ge. These heterostructures exhibit enhanced properties due to synergistic effects, surpassing the capabilities of the individual 2D materials. Notably, heterostructures like TMDs/MXenes,^28^ TMDs/graphene,^29^ MXene/graphene,^30^ graphene/phosphorene,^31^ and germanane/MXene^32^ demonstrate superior energy storage capabilities compared to their constituent units. Integrating nano-Ge into heterostructures with 2D carbon materials can significantly improve intercalation characteristics and ion adsorption sites. Group 14 elements present promising substrates for ZHCs, and recently cyanoethyl-functionalized germanane (Ge–C_2_–CN) has been used as a photocathode for Zn ion storage.^33^ The EC applications of ZHCs utilizing germanane (Ge_6_H_6_ or HGe) and methyl germanane (Ge_6_(CH_6_)_6_ or MGe) in a binder-free architecture remain unexplored.

Herein, we report a versatile method to fabricate ZHC cathodes by in situ functionalizing and cross-linking nano-HGe and MGe with LIG sheets (HGe-LIG/MGe-LIG) by using a single-step lasing process (Scheme 1). The process involves the presynthesis of HGe and MGe by topotactic deintercalation of CaGe_2_ with HCl and methyl iodide (Scheme 1a).^13,34^ Next, we employed a pulsed laser system (diode-pumped Nd:YAG laser) on HGe- and MGe-coated polyimide (PI) sheets. The laser energy induces sufficient mechanical and thermal stress to fragment the HGe/MGe sheets into nanodimensions, while the PI sheet absorbs IR energy, reaching temperatures over 2000 K, and rapidly converts to graphene. The motivation behind this experimental methodology is the formation of stable covalent bonding between the active Ge-based materials and the graphene network. A previously reported article established that laser-based in situ functionalization enables a robust and binder-free architecture where active materials are attached to the current collector via covalent bonding, leading to improved charge transfer and cycling stability.^30,35^ Our experimental design ensures strong attachment between the HGe/MGe and LIG networks and efficient electron–ion transport without any binder or additional conductive agent. Comprehensive material characterization including structural, morphological, optical, and EC analyses is presented to elucidate the fundamental properties of the 2D HGe-LIG and MGe-LIG heterostructures. Subsequently, we compare the ZHC performance of HGe-LIG and MGe-LIG, detailing the Zn^2+^ storage mechanisms and investigating the impact of different functional groups (H/CH_3_) on their EC activity. Moreover, we have utilized both aqueous and organohydrogel electrolytes with dimethyl sulfoxide (DMSO) as an additive to alter the hydrogen-bond network within the hydrogel framework. Density functional theory (DFT) calculations provide insights into the Zn atom adsorption behavior on HGe and MGe surfaces. This study aims to provide a foundation for the development of 2D germanane-based materials in diverse energy storage applications. Future work can explore various terminations and extend these methodologies to other 2D materials in group 14.

**Scheme 1:**
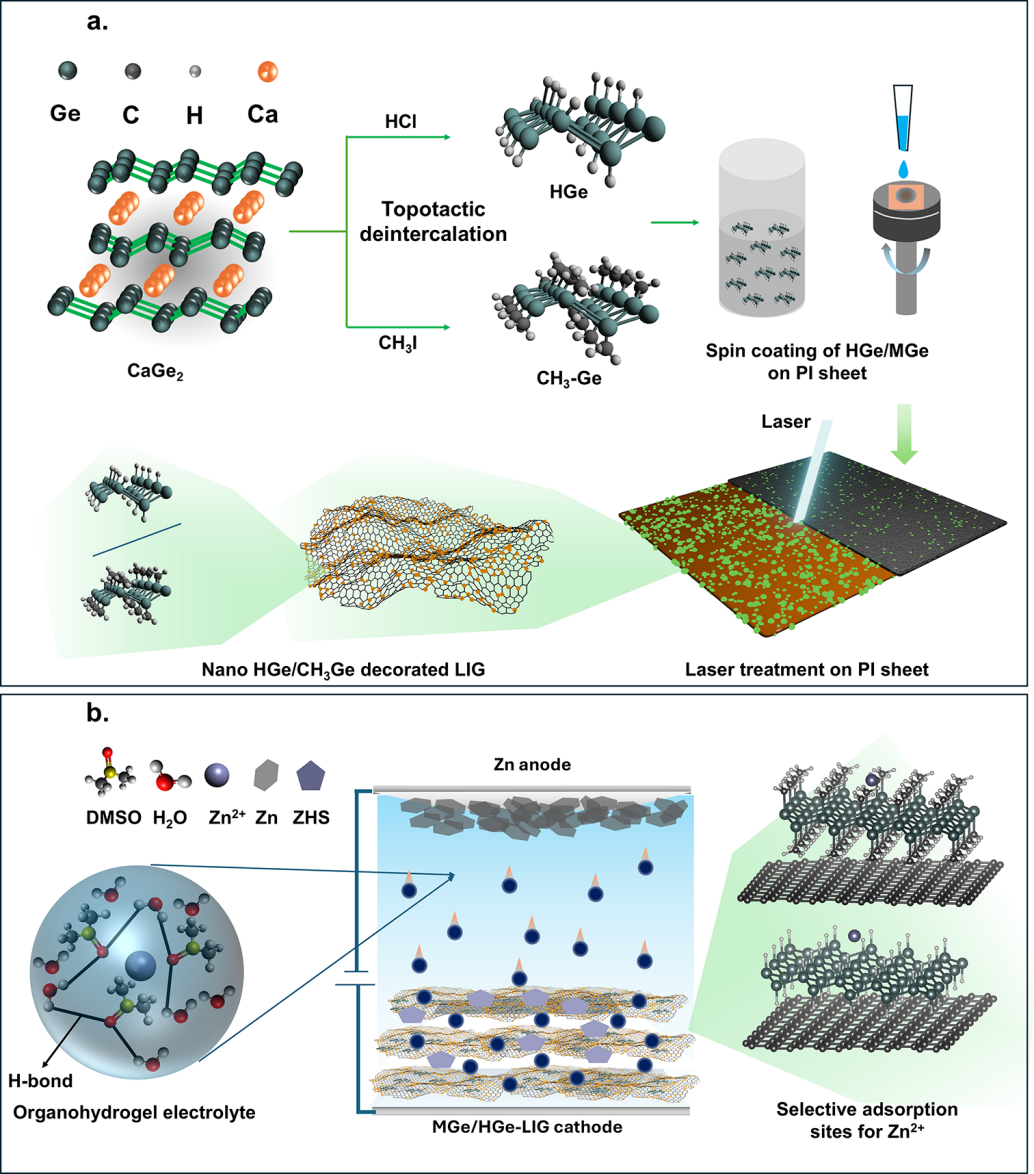
Schematic Diagram of the Workflow: (a) Synthesis of HGe and MGe, Laser Fabrication of HGe/MGe-LIG Hybrid Structure, Stick and Ball Demonstration of HGe/MGe Molecular Structure, and Decoration of HGe/MGe on Graphene Is also Presented; (b) The Assembly of ZHC Device with theTheoretically Simulated Adsorption Insight of Zn^2+^ at Highly Symmetric Sites of Germanane

## Results and Discussions

The scanning electron microscopy (SEM) images reveal the morphological characteristics of the synthesized films and their precursor materials. Both HGe and MGe exhibit a layered structure (marked with a yellow rectangle in Figure S1a and b, Supporting Information). To visualize the individual 2D sheets of HGe and MGe, we carried out transmission electron microscopy (TEM) measurements as well. As shown in Figure S1c, the TEM image of HGe reveals thin 2D layers, where layers are folded into themselves. In contrast, the single 2D sheets of MGe are clearly visible in Figure S1d, where MGe layers have low contrast compared to the TEM grid. At low magnification, the surface morphology of HGe-LIG appeared smoother compared to MGe-LIG (Figure S2, Supporting Information). However, the presence of MGe and HGe is not noticeable in the low-magnification SEM images. The high-magnification SEM (Figure 1a, b) demonstrates the uniform distribution of HGe and MGe across the LIG network. More high magnification further highlights the decoration of nano HGe and MGe sheets on the LIG, with MGe sheets showing a denser arrangement compared to HGe. To see a more clear sheet structure of HGe-LIG and MGe-LIG, low-voltage TEM (operated at 25 keV energy) images were captured (Figure S3, Supporting Information). The resulting low-voltage TEM images of HGe-LIG and MGe-LIG show extended nanosheets at the 100 nm scale bar region. While an overall sheet structure is visible, the sheet surface looks rough for both samples. The roughness appears due to the coating of HGe and MGe nanosheets over LIG sheets. The energy of a short laser pulse of a Nd:YAG pulsed laser beam focused on a nanometric dimension provides high power, which causes sufficient mechanical and thermal stress to break the HGe/MGe sheets into tiny HGe/MGe particles. Simultaneously, the PI sheet absorbs IR energy, quickly reaching temperatures exceeding 2000 K within a submillisecond time scale, which induces the photothermal conversion of the PI sheet into graphene. This single-step method enables the in situ bonding formation between HGe/MGe particles and the LIG network.

**Figure 1:**
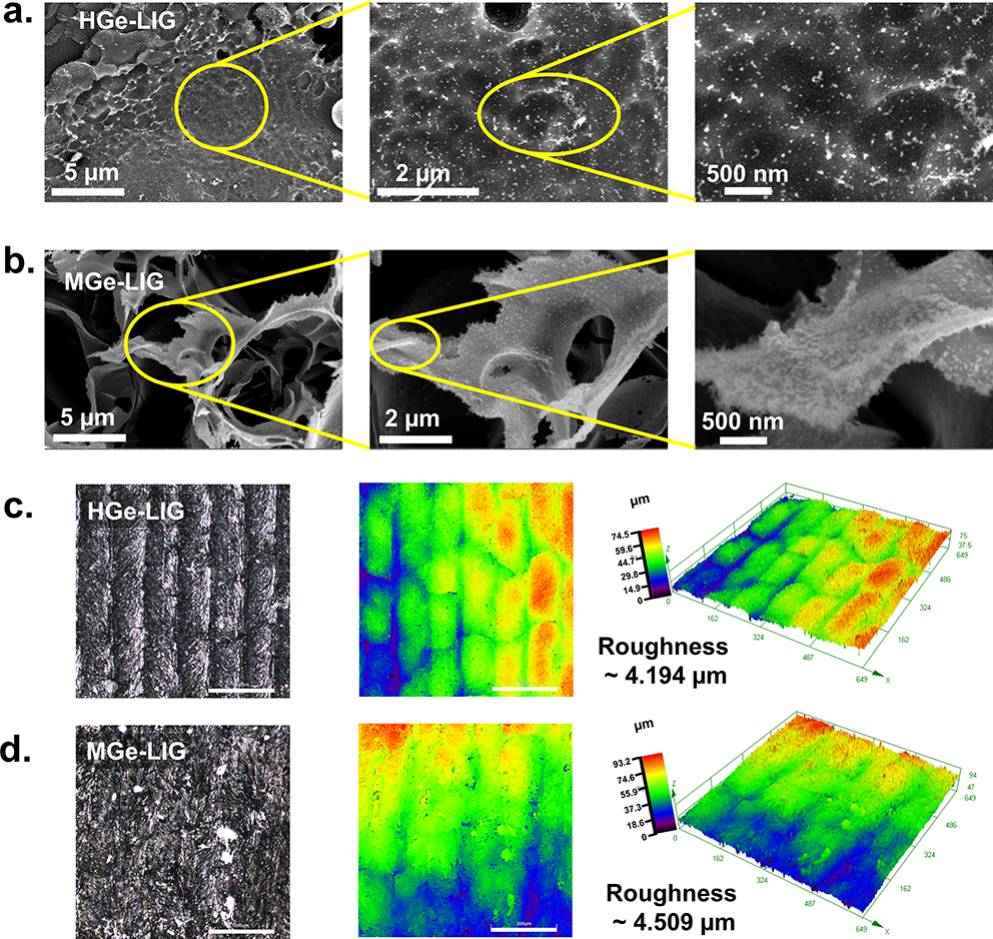
Morphological characterizations. SEM top view of (a) HGe-LIG and (b) MGe-LIG with different magnifications, where uniform decoration of HGe and MGe over LIG is visible. The yellow circles in Figure 1a and 1b highlight the areas captured in the subsequent high-magnification SEM image. CLSM optical images and the corresponding 2D and 3D false-color profiles of (c) HGe-LIG and (d) MGe-LIG. Scale bar: 200 μm.

To confirm the even distribution of HGe and MGe within the LIG sheet, elemental mapping assessment was conducted, with the results presented in Figures S4 and S5 (Supporting Information). The energy-dispersive X-ray spectroscopy (EDX) mapping shows the presence of C, O, and Ge in both HGe-LIG and MGe-LIG films, which is validated further by Raman, X-ray diffraction (XRD), and X-ray photoelectron spectroscopy (XPS) analyses.

To measure the surface roughness, confocal laser scanning microscopy (CLSM) images were acquired for HGe-LIG and MGe-LIG films. The surface features and their 2D and 3D false-color images are presented in Figures 1c and 1d, where height profiles are mapped with distinct color representations. The surface roughness of the HGe-LIG and MGe-LIG was calculated as 4.194 and 4.509 μm, respectively.

We conducted comprehensive material characterization to elucidate the properties of HGe, MGe, and their heterostructures with LIG. Raman spectroscopy confirmed the successful formation of these heterostructures (Figure 2a). The Raman spectrum of HGe showed an in-plane Ge–Ge framework vibration (E_2g_ mode) with a peak at around 284 cm^–1^. For MGe, this peak is blue-shifted to approximately 281 cm^–1^, indicating an increased Ge–Ge–Ge bond angle and a flattened Ge sheet structure. Additional major peaks around 435 cm^–1^ correspond to the symmetric stretching of Ge–O–Ge and Ge–Ge bonds.^36^ Small peaks between 100 cm^–1^ and 170 cm^–1^ were attributed to the complicated translation and rotation of GeO_4_ tetrahedra.^37^ The HGe-LIG spectrum exhibited Ge peaks along with the characteristic graphitic D/G-band. In MGe-LIG, the E_2g_ peak and D/G-band were clearly visible; however, low-intensity peaks were obscured by a strong luminescent background, a common phenomenon in functionalized Ge.^14^

**Figure 2:**
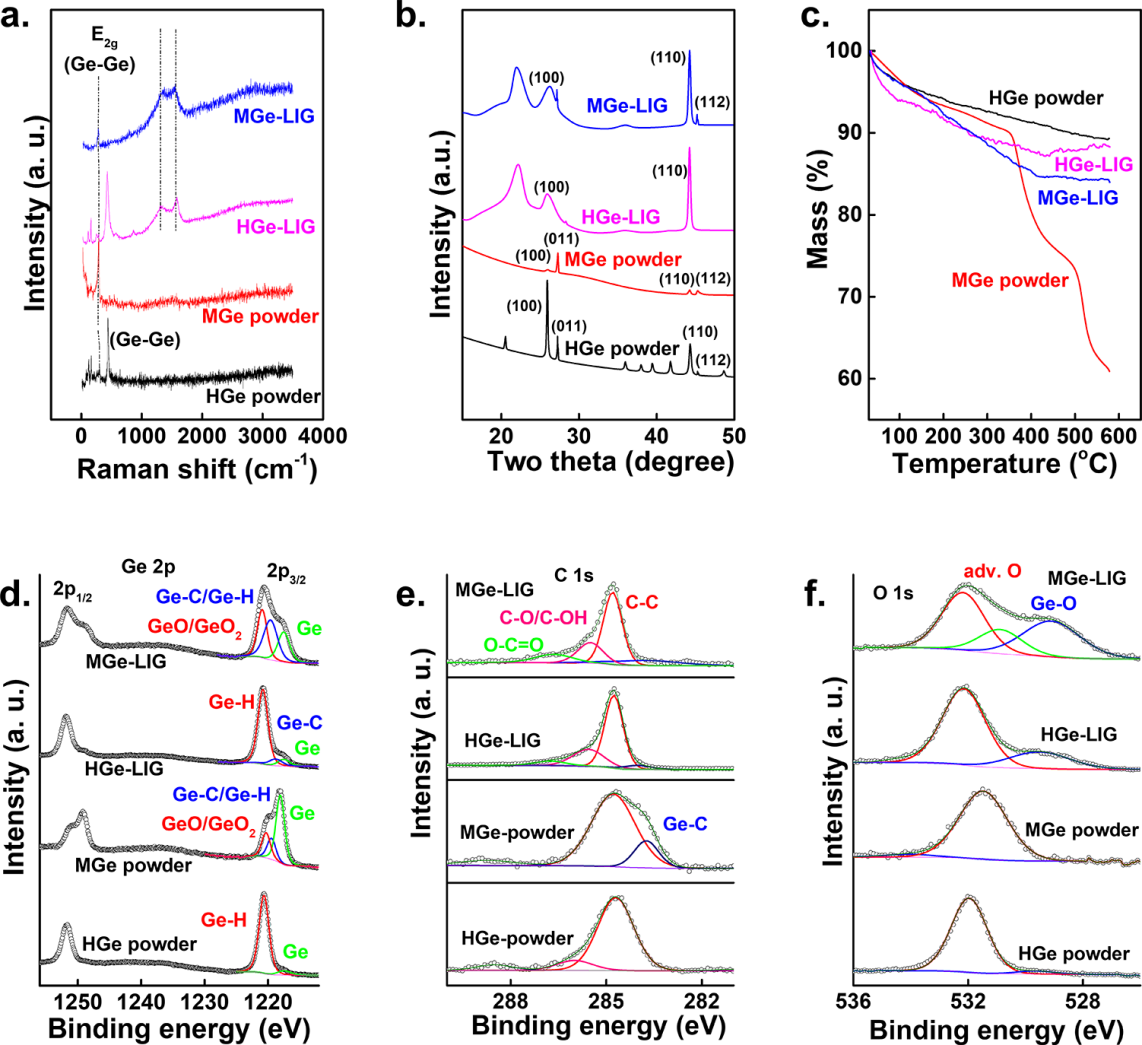
Physical characterizations. (a) Raman spectra, (b) XRD spectra, (c) TGA spectra of HGe/MGe powder and HGe-LIG/MGe-LIG thin films. (d) Ge 2p, (e) C 1s, and (f) O 1s HR XPS spectra of HGe/MGe powder and HGe-LIG/MGe-LIG thin films.

The successful fabrication of the HGe-LIG/MGe-LIG heterostructure was further verified through X-ray diffraction (XRD) analysis (Figure 2b). Both HGe-LIG and MGe-LIG demonstrate broader (100) and (110) XRD peaks as compared to pristine HGe and MGe powders. This broadening suggests that the heterostructured materials predominantly consist of an amorphous phase, particularly in terms of the layer order along the *c*-axis. In atypical hexagonal close-packed structure, the (100) and (110) crystallographic planes represent the structural order along this *c*-axis. The XRD peak broadening and fading suggest that the laser energy interrupts the van der Waals forces, which hold the HGe/MGe layers together. This leads to a loss of long-range order along the *c*-axis. Furthermore, the absence of the (112) peak in HGe-LIG further approves a degree of disorder along the *c*-axis. Due to the loss of orderliness along *c*-axis, only the in-plane lattice parameter (a-parameter) was calculated. The calculated a-parameter remains nearly constant across all materials except for MGe-LIG. The a-parameter is 3.95 Å for HGe, 3.96 Å for MGe, 3.96 Å for HGe-LIG, and 3.91 Å for MGe-LIG. Overall, these a-parameters align well with values previously reported in the literature.^13,14,34^ This transformation from a crystalline to an amorphous structure is advantageous, as the amorphous structure is prone to less mechanical stress and accommodates ions more effectively during intercalation/deintercalation, beneficial for enhancing intercalation-based capacitance.^38,39^

The thermal stability of the prepared films and Ge powders was evaluated by using thermogravimetric analysis (TGA). Both HGe and MGe showed an initial mass loss of approximately 4% when heated from 40 to 100 °C, which is also reflected in HGe-LIG (∼6% mass loss) and MGe-LIG (∼4% mass loss), suggesting the loss of hydrogen or methyl groups (Figure 2c). Beyond 200 °C, HGe exhibited an additional mass loss of about 7%, likely due to the release of chlorine (Cl).^34^ The presence of Cl is evidenced by the elemental XPS analysis. In the case of MGe, significant decomposition happened between 350 and 550 °C, with a mass reduction of ∼35%, attributed to the evolution of Ge­(CH_3_)_2_ due to CH_4_ release.^14^ Conversely, for HGe-LIG and MGe-LIG, the maximum breakdown occurred until 400 °C, with mass losses of ∼13% and ∼15%, respectively. These findings suggest that HGe-LIG and MGe-LIG possess greater thermal stability compared with their HGe and MGe counterparts. The TGA data for LIG is also provided in Figure S6 (Supporting Information) for reference.

Fourier transform infrared spectroscopy (FTIR) in absorption mode confirms the surface termination of the methyl group (CH_3_) in MGe (Figure S7, Supporting Information). For HGe, the vibrational modes between 780 and 855 cm^–1^ were observed. These modes originate from Ge–H_2_ bond bending, which was previously observed in HGe.^34^ The major modes corresponding to −CH_3_ stretching (∼2910 cm^–1^), −CH_3_ bending (∼1400 cm^–1^ and ∼1236 cm^–1^), and −CH_3_ rocking (∼772 cm^–1^) were observed in MGe.^13^ The shoulder peak (within the range of 800 to 1000 cm^–1^) of −CH_3_ rocking signifies the presence of Ge–O bonding vibration^40^ and the signal around 1600–1700 cm^–1^ relates to the bending vibrational mode of O–H bonds. It is important to note that neither HGe-LIG nor MGe-LIG displays the distinct FTIR characteristic peaks of HGe and MGe, except for a faint peak around 865 cm^–1^, which corresponds to GeO_*x*_.

To confirm the surface functionalization and elemental composition, XPS measurements were carried out. The high-resolution (HR) XPS spectra of HGe, MGe, HGe-LIG, and MGe-LIG are presented in Figure 2d–2f. The remaining survey spectra are included in the Supporting Information (Figure S8). Note that the materials exhibited significant surface charging during the measurements. This was mitigated by charge compensation and energy correction to adventitious carbon (284.8 eV). Consequently, the chemical shifts of individual species in the HR spectra cannot be assigned with absolute certainty. Therefore, rather than facilitating direct comparison among different terminations, these analyses primarily provide insights into the structural characteristics of the materials. XPS survey spectra reveal traces of impurities, Ca and Cl (Table S1 Supporting Information). The Ca 2p peak at binding energy (BE) 343 eV can be assigned to Ge–Ca, and its origin is attributed to the synthesis of germanane from calcium germanide (CaGe_2_).^34,41^

We present the HR Ge 2p (Figure 2d), C 1s (Figure 2e), and O 1s (Figure 2f) spectra of HGe/MGe and their heterostructures with LIG. The Ge 2p core level spectra of HGe and HGe-LIG are deconvoluted into three peaks: (i) elemental germanium (∼1217.5 eV, green line), (ii) Ge–C (∼1219.7 eV, blue line), and (iii) Ge–H (∼1220.7 eV, red line). Whereas, for MGe and MGe-LIG, the Ge 2p spectra are deconvoluted into (i) elemental germanium (∼1217.5 eV, green line), (ii) Ge–H/Ge–C (∼1219.7 eV, blue line), and (iii) Ge–O and Ge–O_2_ (∼1220.9 eV, red line), originating from the surface oxidation of germanane. The HR XPS Ge 2p spectra of HGe-LIG show no sign of oxidation, with the spectra dominated by Ge–H/Ge–C. This protection of elemental Ge is crucial to Zn^2+^ intercalation. Whereas MGe-LIG displays partial surface oxidation, with an enhancement of GeO_*x*_ as compared to pristine MGe powder. The surface oxidation likely appears due to the partial decomposition of methyl groups during laser irradiation. While this slight oxidation plays a key role in Zn^2+^ storage performance, it significantly decreases the sheet resistance of the material. To validate this, we measured the sheet resistance of both HGe-LIG and MGe-LIG films using a four-point probe. Based on six measurements, the average sheet resistance value was calculated as 42.78 ± 3.5 Ω square^–1^ and 88.64 ± 5.6 Ω square^–1^ for HGe-LIG and MGe-LIG, respectively

The presence of carbon-containing substituents (Ge–C) in HGe, MGe, HGe-LIG, and MGe-LIG was confirmed by a distinct shoulder peak at ∼282.5 eV in the C 1s spectra (blue line in Figure 2e). Note that at % of these substituents is maximum in MGe/MGe-LIG. In addition, the peaks at BEs of ∼286.6 and 288.6 eV corresponding to C–O/C–OH and O–C=O are also observed (C 1s spectrum, pink line and green line).

Most of the oxygen groups are located in the higher BE region, indicative of adventitious contamination of the samples. The presence of oxides or hydroxides (Ge–O) is inferred from the shoulder peak at approximately 530 eV, which is particularly prominent in the O 1s spectra for MGe-LIG.

The in-depth materials characterization evidences the successful attachment of HGe and MGe over the LIG sheet and provides a fundamental understanding of the heterostructure. Next, we investigated the comparative EC performance of the HGe-LIG/MGe-LIG cathode in different electrolytes.

### Electrochemical Analysis of ZHCs in Aqueous Electrolyte

As a proof of concept, ZHCs were assembled by using Zn foil as the anode, HGe-LIG/MGe-LIG as the cathode, and 2 M ZnSO_4_ as the aqueous electrolyte (Figure 3j). The cyclic voltammetry (CV) curve of HGe-LIG//Zn and MGe-LIG//Zn cells exhibited similar shapes, while the HGe-LIG//Zn showed an improved CV integration area as compared to MGe-LIG//Zn (Figure S9, Supporting Information). The capacitive- and diffusion-controlled contributions to the charge storage were quantified based on the CV analyses. Figure 3a and 3d represent CV curves of MGe-LIG and HGe-LIG electrodes at different scan rates. Two pairs of redox peaks were observed, labeled as peaks i and ii for the anodic processes and peaks iii and iv for the cathodic processes. CV curves of both HGe-LIG and MGe-LIG cathodes at varying scan rates still hold the typical rectangular profiles, indicating a high capacitive nature. The surface capacitive effect of the electrode was calculated using the equation *i* = *av* ^*b*^ , where *i* is the measured current, *v* is the scan rate, and *a* and *b* are the empirical factors.^42,43^ The *b* values were derived from the slope of the plot of log *i* versus log *v*, according to the following equation: log *i* = log *a* + *b* log *v*. The *b* value close to 0.5 indicates a faradaic intercalation-dominant charge storage process, while the *b* value approaching 1 corresponds to the capacitive-dominant response. The calculated *b* values for the redox peaks (i) to (iv) for both MGe-LIG and HGe-LIG are close to 1 (Figure 3b and 3e) suggesting that the Zn^2+^ kinetics are mainly surface-controlled. Furthermore, the surface capacitive behavior and diffusion contribution were quantified by separating current response *i* at the constant potential *V,* as defined by equation *i* (V) = *k*_1_ *v* + *k*_2_ *v* ^1/2^, where *k*_1_ *v* represents the surface capacitive effects and *k*_2_ *v* ^1/2^ represents the diffusion-controlled process.^44^ With increasing scan rates from 0.2 to 1 mV s^–1^, the surface-controlled contribution to the overall charge storage rises from ∼60% to ∼80% for both MGe-LIG and HGe-LIG electrodes (Figure 3c and 3f). To further visualize this, a representative CV profile at 1 mV s^–1^ is displayed as Figures S10 and S11 (Supporting Information) where the capacitive current (highlighted in red) clearly dominates the total current for both electrodes.

**Figure 3:**
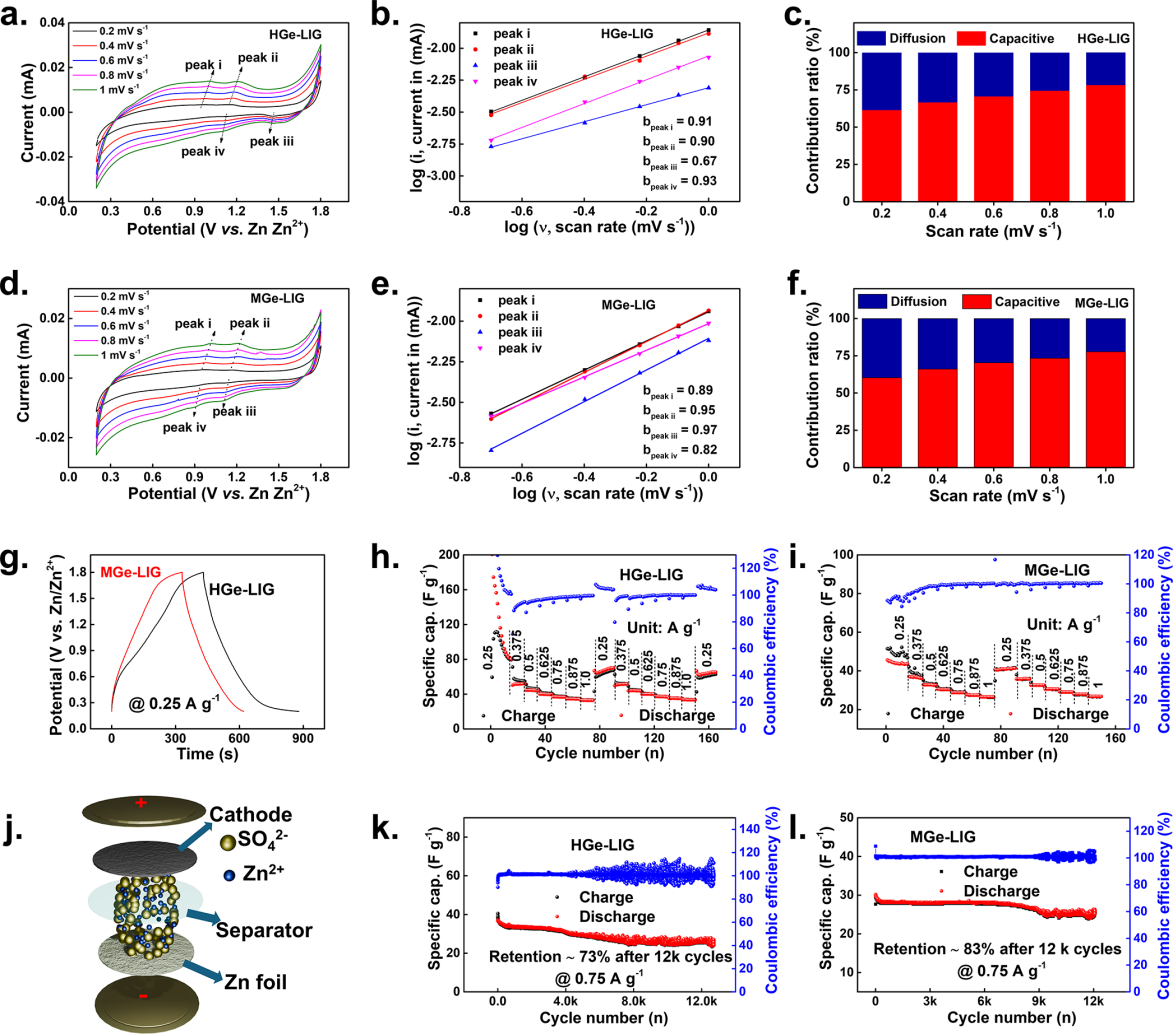
Electrochemical performance of HGe-LIG and MGe-LIG cathode-based ZHCs in 2 M ZnSO_4_ aqueous electrolyte. (a, d) CV profiles of the HGe-LIG and MGe-LIG cathodes at different scan rates. (b, e) log *i* vs log *v* plots of anodic and cathodic peaks based on the CV profiles. (c, f) Contribution ratios of the capacitive and diffusion components in HGe-LIG and MGe-LIG cathodes. (g) GCD comparison between the HGe-LIG and MGe-LIG cathodes. Rate performances of (h) HGe-LIG and (i) MGe-LIG cathodes at varying current densities. (j) Schematic diagram of the ZHC device. Cyclic stability performances of (k) HGe-LIG and (l) MGe-LIG at a current density of 0.75 A g^–1^.

Being consistent with the CV curve, the discharge time in the galvanostatic charge–discharge (GCD) profile becomes prolonged for HGe-LIG//Zn as compared to MGe-LIG//Zn (Figure 3g). Notably, HGe-LIG displayed a high gravimetric capacitance of ∼104 F g^–1^ at the current density of 0.25 A g^–1^, while the MGe-LIG showed ∼46 F g^–1^ respectively. Figure S12 (Supporting Information) presents a comparative GCD profile of pristine LIG, HGe-LIG, and MGe-LIG, clearly demonstrating the superior performance of HGe-LIG and MGe-LIG over LIG, as evidenced by their extended discharge times. The GCD comparison clearly shows that both HGe-LIG and MGe-LIG electrodes deliver more than 4 times higher capacity than the LIG electrode alone. This significant improvement directly highlights the dominant contribution of the active materials (HGe/MGe) once they are attached to the LIG component. Both HGe-LIG//Zn and MGe-LIG//Zn ZHCs exhibited outstanding rate capabilities at current densities from 0.25 to 1 A g^–1^, respectively (Figure 3h and 3i). The Coulombic efficiencies (CE) of the initial dozen cycles were moderately high for HGe-LIG//Zn ZHCs. Apart from that, the CE maintained ∼100% during the whole process. Interestingly, the MGe-LIG//Zn ZHC offers improved cycling stability (∼83% capacitance retention after 12000 cycles) as compared to HGe-LIG//Zn ZHC, which retains only ∼73% capacitance retention after 12000 cycles (Figure 3k, 3l). Overall, the HGe cathode is promising for delivering high capacities, whereas for long-term stability in an aqueous solution, covalent CH_3_ termination on Ge would be a better option. We have added a comparison (Table S4, Supporting Information) of our device performance with other state-of-the-art literature reported recently.

### Charge Storage Mechanism

To explain the charge storage mechanism of the HGe-LIG and MGe-LIG cathodes, a combination of ex situ XRD and XPS measurements was performed. Figure 4a and Figure 4e display the discharge/charge profiles of HGe-LIG and MGe-LIG for the third cycle, where marked points (states i to vi) were picked for the ex situ XRD and XPS measurements.

**Figure 4:**
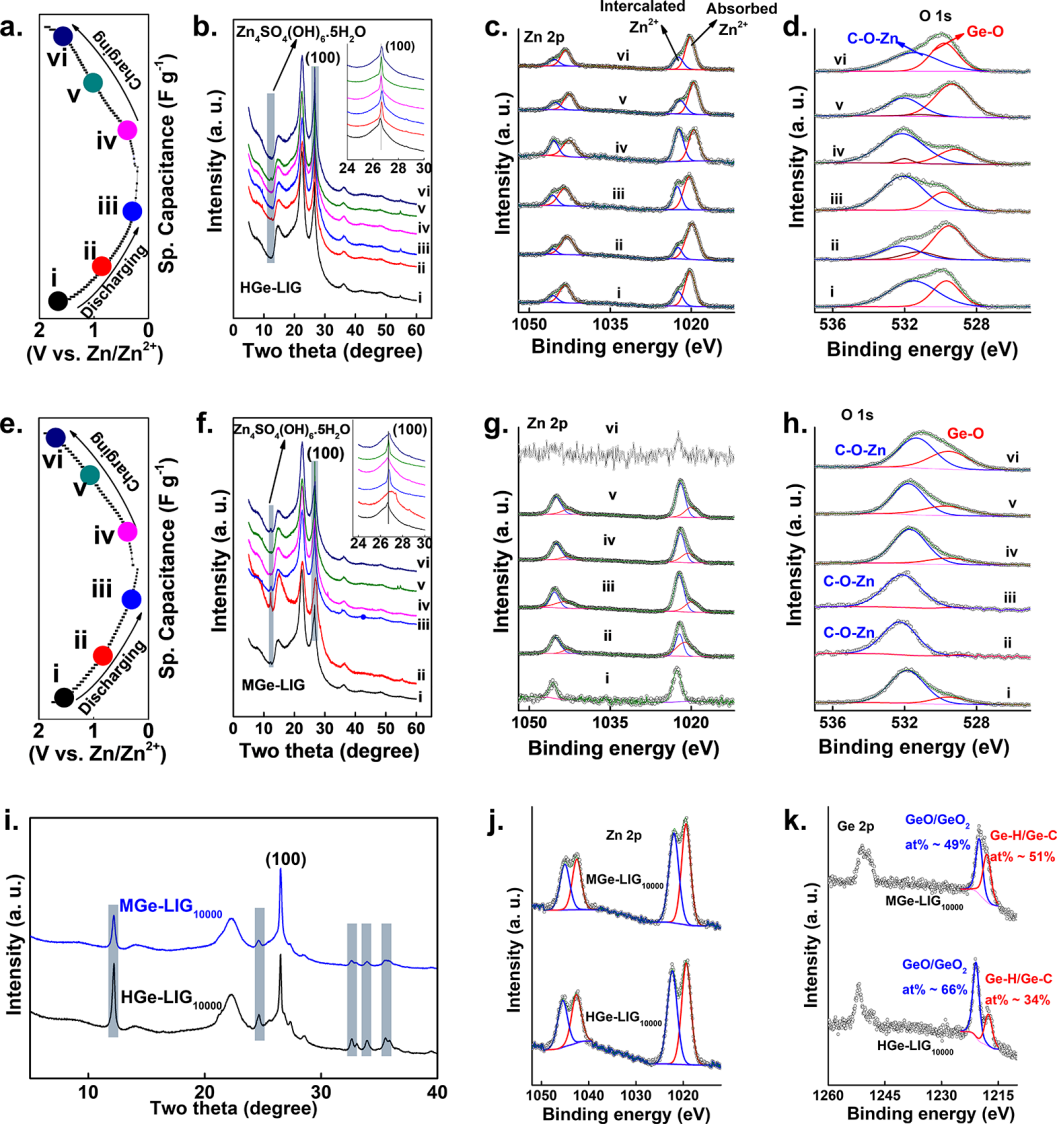
Charge storage mechanism of HGe-LIG//Zn and MGe-LIG//Zn ZHCs. (a) Discharge/charge profile of the HGe-LIG cathode. (b) Ex situ XRD spectra of the HGe-LIG cathode at various states of the discharge/charge process. The inset represents the zoomed view within the region of 2θ ∼ 24–30 degrees. Ex situ XPS spectra of the HGe-LIG cathode focus on the (c) Zn 2p region and (d) O 1s region. (e) Discharge/charge profile of the MGe-LIG cathode. (f) Ex situ XRD spectra of the MGe-LIG cathode at various states of discharge/charge process. The inset represents the zoomed view within the region of 2θ ∼ 24–30 degrees. Ex situ XPS spectra of MGe-LIG cathode focusing on the (g) Zn 2p region and (h) O 1s region. (i) XRD spectra of HGe-LIG and MGe-LIG after 10000 GCD cycles. The highlights represent the ZHS peaks. (j) Zn 2p and (k) Ge 2p HR XPS spectra of HGe-LIG and MGe-LIG after more than 10000 GCD cycles.

During XRD analysis of the HGe-LIG cathode, a negligible shift of the 100 peak position, along with the appearance of a diffraction peak of Zn_4_SO_4_(OH)_6_·5H_2_O (ZHS, JCPDS # 44-0673) is observed at 12.26° (Figure 4b, plots ii and iii) during the discharging process.^45^ The ZHS peak gradually disappears with a shift of the 100 peak to a higher 2θ value during the subsequent charging process (Figure 4b, plots v and vi). These results indicated the intercalation of Zn^2+^/H^+^ and the formation of the basic zinc salt ZHS due to the cathode uptake of H^+^ from the electrolyte as the potential continuously decreased from 1.6 to 0.2 V. Reversibly, during charging, the ZHS phase vanished as the potential ramped, indicating the dissolution of the adsorbed zinc salt. This also means that the byproducts of ZHS are unlikely to deteriorate the active sites in the HGe-LIG cathode. This reversible adsorption/desorption or intercalation/deintercalation process was further verified by ex situ XPS measurements. As observed in Figure 4c, the Zn 2p XPS scan revealed two pairs of peaks (1020.25/1022.35 eV and 1043.35/1045.75 eV for Zn_3/2_ and Zn_1/2_, respectively) at the beginning of the discharge cycle. The signal at ∼1021 eV can be assigned to the absorbed Zn salt on the cathode surface, whereas the peak at a higher BE of ∼1023 eV corresponds to the intercalated Zn^2+^.^46^ When the cathode was gradually discharged to 0.2 V, the intercalated peak strengthened intensity. During the subsequent charging process, the intercalated peak gradually loses intensity, presenting the reversible Zn^2+^ exchange in the HGe-LIG cathode. The ex situ XPS O 1s (Figure 4d) spectra showed a gradual increase and decrease of the C–O–Zn peaks during the discharging and charging processes. This further supports the reversible absorption/desorption of intercalation/deintercalation of Zn^2+^ on the HGe-LIG cathode. Note that the formation of the ZHS layer oxidizes Ge; as a result, the Ge 2p state is mostly dominated by the Ge–O/Ge-O_2_ state during the discharge/charge cycle (Figure S13, Supporting Information).

For MGe-LIG, a similar trend of XRD peak (Figure 4f) was observed, except that the ZHS peak is more prominent than in HGe-LIG. The prominent appearance of the ZHS peak (2θ ∼12.25°) upon charging, along with the dissolution of Zn_4_SO_4_(OH)_6_·5H_2_O at higher potential is demonstrated by the gradual decline in intensity. Note that the ZHS peak did not completely vanish when the potential ramped from 0.2 to 1.6 V, indicating incomplete dissolution of adsorbed zinc salt when the cathode releases H^+^. The typical electron-donating nature of methyl functional groups could be the reason for the incomplete removal of ZHS. Therefore, Zn deposition/stripping became an exclusive storage mechanism once the hysteretic Zn_4_SO_4_(OH)_6_·5H_2_O phase was formed. This process was further observed from ex situ XPS survey. The typical pairs of peaks of the Zn 2p state (1022.35/1045.65 eV) were observed at the beginning of the third discharge cycle (Figure 4g) corresponding to the surface-absorbed Zn^2+^. With gradual discharge to 0.2 V, a new pair of peaks appeared, resulting from the continuous diffusion of Zn^2+^ in the MGe-LIG cathode.^46–48^ These peaks vanished during the successive charging process, indicating the favorable reversibility of Zn^2+^. Besides, the strengthening of the intensity of C–O–Zn during discharging and the progressive weakening of C–O–Zn upon charging in the O 1s region further validate the reversible absorption/desorption of Zn^2+^ on the electrode surface (Figure 4h). A reversible evolution of the Ge 2p (Ge–O or Ge–O_2_/Ge–C or Ge–H) peak through an intermediate germanane oxide (GeO or GO_2_) is also visible during the gradual discharging and charging process (Figure S14, Supporting Information).

The interaction between hydrated Zn^2+^ ions and the HGe-LIG/MGe-LIG surface is additionally evidenced by in situ Raman measurements, which reveal a reversible change in the D and G band intensities with potential (Figure S15, Supporting Information). This trend is consistent with earlier reports on nanoporous carbon and spectral variations with negative doping and metal-ion–graphene coupling,^49,50^ confirming that hydrated Zn^2+^ ions are electrochemically adsorbed onto the carbon surface.

To investigate the reasons behind the superior stability of MGe-LIG compared to HGe-LIG after 10000 charge–discharge cycles, we conducted ex situ XRD and XPS analyses of the cathodes, designated as HGe-LIG_10000_ and MGe-LIG_10000_. The XRD patterns (Figure 4i) for both samples revealed distinct ZHS peaks at 12.18°, 24.64°, 32.62°, 33.94°, and 35.88° (JCPDS # 44–0673). Notably, the ZHS peaks were more pronounced in the HGe-LIG_10000_ sample, suggesting significant formation of a Zn salt layer at the cathode–electrolyte interface. This observation was further supported by XPS analysis in the Zn 2p region (Figure 4j), which indicated the simultaneous presence of the absorbed Zn salt and intercalated Zn.

However, these findings alone did not fully explain the enhanced stability of MGe-LIG_10000_ over that of HGe-LIG_10000_. To gain deeper insights, we performed ex situ XPS analysis at the Ge 2p edge. The Ge 2p spectra showed a clear split, where MGe-LIG_10000_ underwent less progressive oxidation during extended cycling compared to that of HGe-LIG_10000_. This reduced oxidation in MGe-LIG_10000_ during the charging process, marked by Zn ion deintercalation and electron loss at the cathode, allows MGe-LIG to undergo less structural degradation, contributing to its superior long-term stability. The XPS analysis in the Ge 2p region before and after long cycles is presented in Table S2 (Supporting Information).

To explain MGe-LIG’s superior stability, we further carried out cycle-resolved electrochemical impedance spectroscopy (EIS) measurements until 5000 cycles (Figure S16, Supporting Information). MGe-LIG’s superior stability (83% retention at 12,000 cycles vs 73% for HGe-LIG) implies slower charge transfer resistance (*R*_ct_) growth. This is attributed to the methyl-induced electron donation, reducing Zn nucleation overpotential and Ge oxidation. Cycle-resolved EIS confirms MGe-LIG’s lower Δ*R*_ct_ (Table S3, Supporting Information), directly evidencing stabilized charge transfer.

Based on the above ex situ characterizations, we can say that the charge storage mechanism of HGe-LIG and MGe-LIG cathodes in aqueous electrolyte (2 M ZnSO_4_) is controlled by intercalation of Zn^2+^, physical adsorption/desorption, reversible chemical adsorption/desorption, and continuous diffusion of Zn^2+^ ions at the favorable sites of HGe/MGe molecules. To understand the adsorption behavior of Zn atoms on the HGe and MGe, we have carried out theoretical calculations.

### Theoretical Insights into Zn Adsorption on HGe and MGe Surfaces

DFT computations were executed to understand the adsorption of a Zn atom on HGe and MGe. The 2D slabs of the respective materials were constructed as periodic models with a thickness of 23 Å to simulate the 2D structures. These models featured Ge–Ge, Ge–Me, and Ge–H bond lengths of 2.5 Å, 2.0 Å, and 1.6 Å, respectively, as depicted in Figure 5. The Zn atom was evaluated for adsorption at three highly symmetric sites of germanene, i.e., top (t), bridge (b), and hollow (h) sites (Figure 5). At full methyl (Me) and hydrogen (H) coverage of germanene, the Zn atom was preferentially bound above the top site and between the three functional groups (either Me or H). The adsorption energies were calculated to be −0.21 eV and −0.30 eV, respectively, with Zn–Me and Zn–H distances of 3.2 and 2.8 Å (Figures 5b and 5f). When Zn was adsorbed above the Me group or H adatom, the adsorption was slightly lower, with adsorption energies of −0.15 eV and −0.17 eV, respectively, and the Zn–Me and Zn–H distances of 3.1 and 2.5 Å, respectively (Figure 5c and Figure 5g). Removal of the Me group or the H adatom from germanane caused higher adsorption of Zn directly to the Ge atom at the top site, with adsorption energies of −0.87 and −0.77 eV and the Zn–Ge bond lengths of 2.7 and 2.6 Å, respectively (Figure 5d and Figure 5h). In all simulations, Zn spontaneously moved from the initial bridge site to the top one. The Zn adsorption in the hollow site of MGe and HGe was weaker, with adsorption energies of −0.13 eV and −0.31 eV for Zn@MGe and Zn@HGe and the Zn–Me and Zn–H distances of 3.0 Å and 2.8 Å, respectively. Interestingly, the removal of either the Me group or the H adatom caused the emergence of magnetic character due to the imbalance of two sublattices of germanene^51^ as was observed also in other 2D materials,^52,53^ for example, in partially fluorinated/defluorinated graphene.^54^

**Figure 5:**
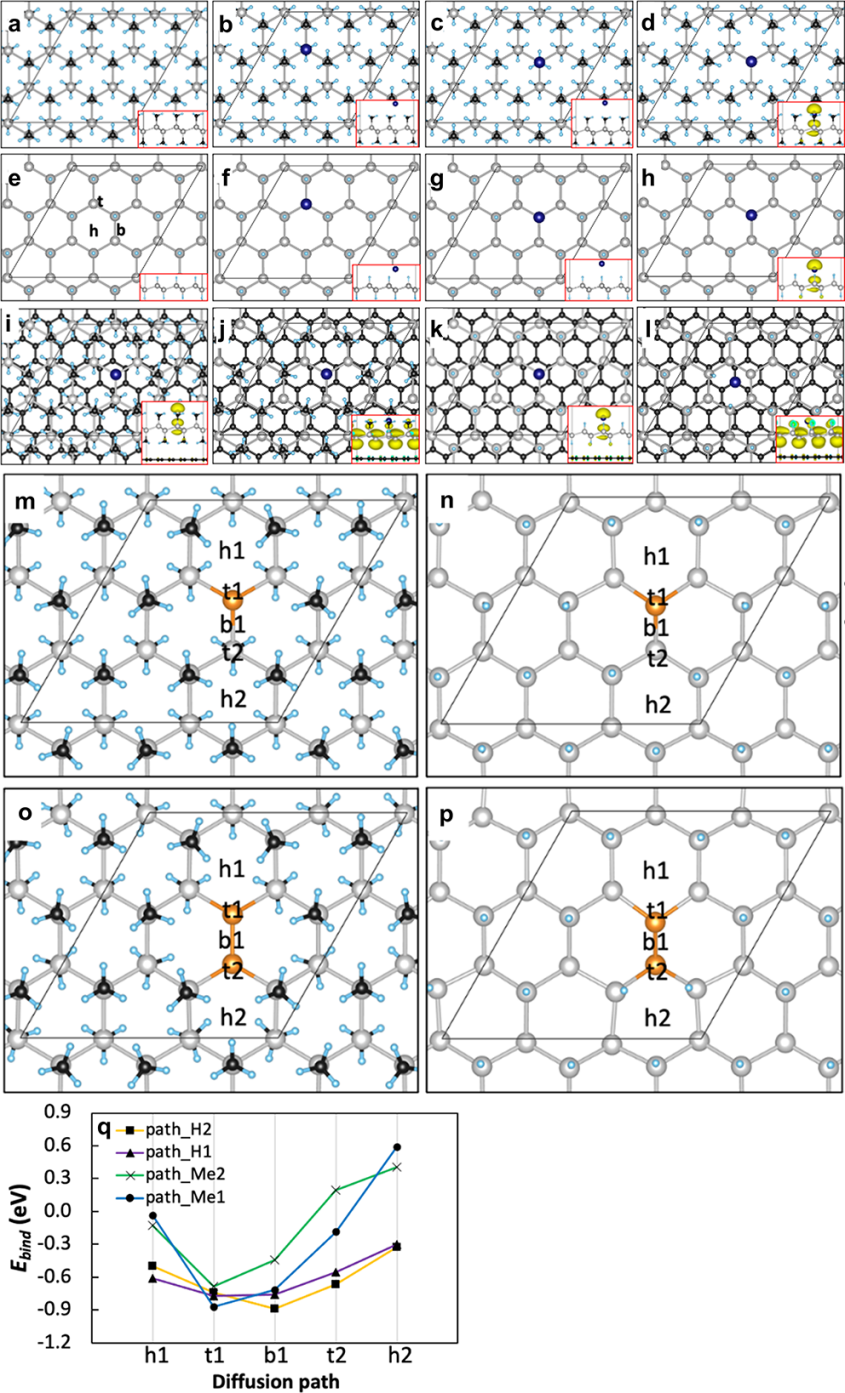
DFT calculation reveals the possible adsorption sites of Zn and possible diffusion path of Zn. Optimized models of (a) MGe, (b–d) Zn@MGe, (e) HGe, (f–h) Zn@HGe, (i–j) Zn@MGe/graphene, and (k–l) Zn@HGe/graphene. Germanene atoms are shown as silver spheres, hydrogen in light blue, carbon in black, and zinc in dark blue. The t, b, and h letters represent top, bridge, and hollow sites, respectively. Positive/negative spin density distribution is shown in yellow/cyan (isosurface ±0.0025 au). (m, o) Panels show models and the diffusion path of Zn on MGe. (n, p) Panels show models and the diffusion path of Zn on HGe. (q) Binding energies of Zn adsorbed at highly symmetric sites of hydrogenated and methyl-terminated germanene. The t, b, and h letters represent top, bridge, and hollow sites, respectively. As for MGe and HGe, we considered one (m, n) and two (o, p) removed Me groups or H adatoms, where sp^2^ Ge atoms are colored in orange.

To elucidate the effect of graphene on MGe and HGe, we placed the optimized model, which possessed the highest adsorption of Zn on the graphene layer (Figure 5i and Figure 5k) and single-sided functionalized MGe and HGe on the graphene layer (Figure 5j and Figure 5l). While adsorption energies and the Zn–Ge bond lengths of MGe/graphene and HGe/graphene remained unchanged as compared to the freestanding MGe and HGe, the adsorption energies of Zn decreased at the single-sided functionalized MGe/graphene and HGe/graphene, i.e., from −0.87 eV to −0.57 eV for MGe/graphene and from −0.77 eV to −0.65 eV for HGe/graphene, which could be assigned to the structural changes and higher magnetic moment of Zn@MGe/graphene and Zn@HGe/graphene (7.6 μ_B_ vs 1 μ_B_). Graphene–MGe and graphene–HGe interacted at 2.8–3.5 Å distance, indicating dispersion interaction and π–π stacking (Figure 5i−l).

While fully methyl-terminated and hydrogenated germanane were shown as nonmagnetic semiconductors with band gaps of 0.9 and 1 eV, respectively (Figure S17a and b, Supporting Information), Zn@MGe and Zn@HGe with one removed functional group possessed a semiconducting electronic structure with localized states at the Fermi level (Figure S17c and d, Supporting Information), which indicated the magnetic character of the material. The graphene substrate lowered the band gap of Zn@MGe/graphene and Zn@HGe/graphene, indicating higher conductivity of the material (Figure S17e and f, Supporting Information). The density of states (DOS) of single-sided functionalized Zn@MGe/graphene and Zn@HGe/graphene DOS showed more localized states at the Fermi level due to a higher spin polarization of the system by the sublattice imbalance of germanene (Figure S17g and h, Supporting Information).

The diffusion of Zn on MGe and HGe surfaces was monitored along the h_1_–t_1_–b_1_–t_2_–h_2_ pathway, with optimized Zn height above the high-symmetry positions (Figure 5m–p). As for MGe and HGe, we considered one (Figure 5m and Figure 5n) and two (Figure 5o and Figure 5p) removed Me groups or H adatoms. In all systems, the Zn atom tended to move from the hollow and bridge sites to the top one. Note that although Zn was placed above the flat b_1_ site, the Zn atom was bonded to one Ge atom due to the buckled honeycomb structures of germanane after the optimization, i.e., rather on top of Ge instead of the bridge site. The BE profile of Zn along HGe was shallower as compared to Zn@MGe, suggesting a more facile Zn diffusion above the HGe surface, in agreement with experimental long-term stability and retention measurements.

### Organohydrogel-Based ZHCs

In this study, ZHCs were assembled as a proof-of-concept using zinc foil as the anode, HGe-LIG or MGe-LIG as the cathode, and polyacrylamide (PAM) hydrogel and organohydrogel as the electrolyte. Note that the PAM gel served dual roles as the electrolyte and the separator.

While using PAM-based hydrogel electrolyte, the GCD profiles showed slightly longer discharge times for HGe-LIG//Zn (490 s) compared to MGe-LIG//Zn (469 s; Figure S18, Supporting Information) ZHCs. Hence, all subsequent measurements were conducted exclusively using the HGe-LIG cathode. The ZHCs using PAM hydrogel electrolyte with HGe-LIG//Zn exhibited a maximum capacitance of approximately 38 F g^–1^ at a current density of 0.25 A g^–1^ and excellent rate performance (Figure S19, Supporting Information) with varying current density.

To improve the capacity of hydrogel-based ZHCs, we have added dimethyl sulfoxide (DMSO) to hydrogel electrolytes, which is capable of suppressing the hydrogen evolution reaction and zinc dendrite formation.^55–58^ Considering these benefits, we synthesized a PAM organohydrogel electrolyte in a DMSO/H_2_O binary solvent system, which retained the transparency of the PAM hydrogel electrolyte (Figure S20, Supporting Information). ZHCs were then assembled with the PAM organohydrogel electrolyte (Figure 6a) without using any separator. Interestingly, the GCD period for HGe-LIG//Zn significantly increased from 490 s in the hydrogel to 1013 s in the organohydrogel electrolyte (Figure 6b). The specific capacitance of the HGe-LIG cathode improved from 38 F g^–1^ with the hydrogel electrolyte to 87 F g^–1^ with the organohydrogel electrolyte at a current density of 0.25 A g^–1^. The HGe-LIG//Zn ZHCs demonstrated remarkable rate capability, with discharge capacities quickly recovering upon returning to lower current densities, indicating excellent structural stability and substantial tolerance to Zn^2+^ intercalation/deintercalation or adsorption/desorption processes (Figure 6c). The scaling-up HGe-LIG//Zn ZHC device was assembled into a pouch cell configuration (Figure 6d). The GCD curve confirms that the HGe-LIG//Zn pouch cell delivers a specific capacitance of 16.7 F g^–1^ at 1.35 A g^–1^ (Figure 6e). Besides, the discharge time remains the same for the HGe-LIG//Zn pouch cell in the bending state (Figure S21, Supporting Information). Particularly notable was the ultralong cyclic stability exhibited by the scaling-up device, maintaining reversible capacity (∼94% retention) after 10000 charge/discharge cycles (Figure 6e), suggesting robust structural integrity over long-term cycling. To operate small electronic devices, we connected a pouch cell in series, enabling a higher output voltage and current (inset of Figure 6e). When configured this way, the combined voltage window increases to ∼3.6 V, while the discharge duration remains nearly identical to that of a single pouch cell under the same current density (Figure S22, Supporting Information).

**Figure 6:**
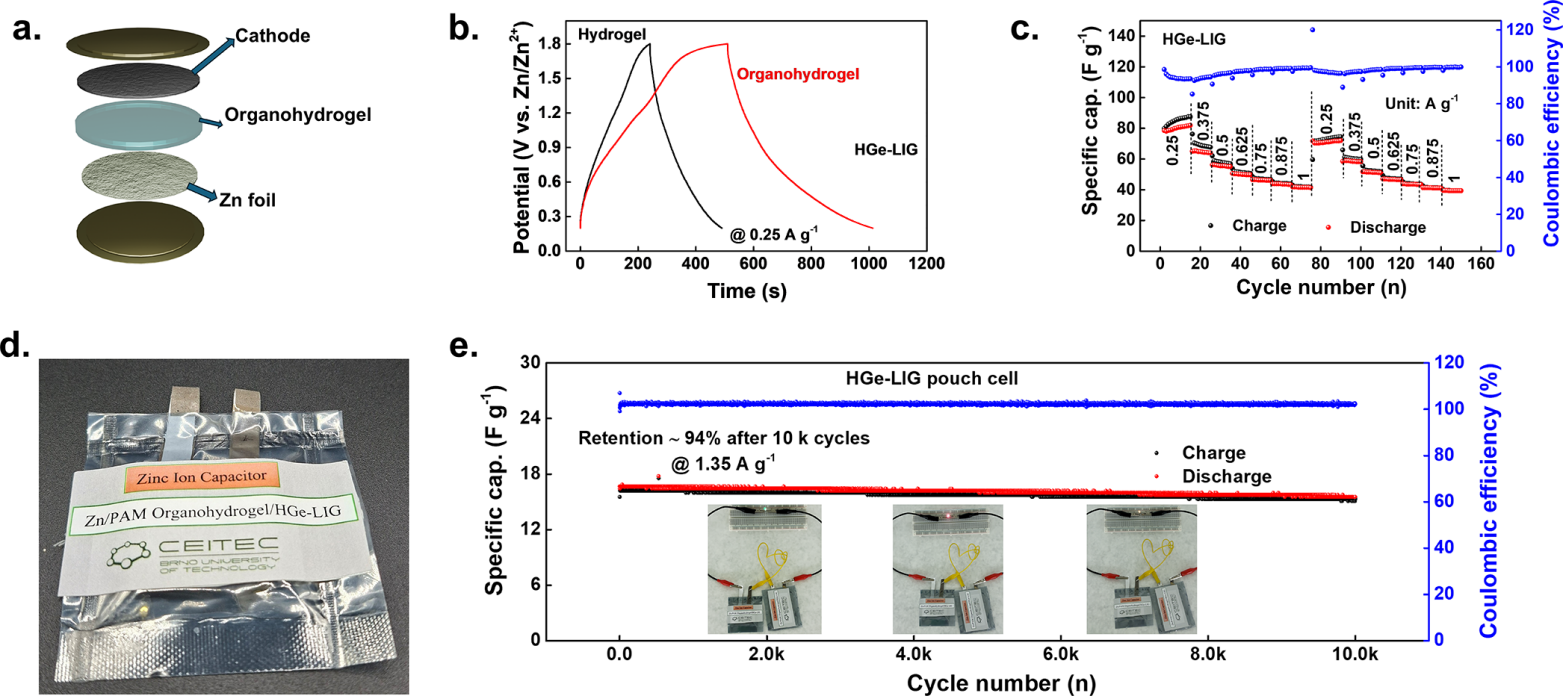
Electrochemical performance of the HGe-LIG cathode-based ZHC in organohydrogel electrolyte. (a) Schematic diagram of the ZHC device where organohydrogel acts as both the electrolyte and the separator. (b) Comparison of GCD profiles for HGe-LIG cathodes in hydrogel and organohydrogel electrolytes. Rate performances of the (c) HGe-LIG cathode at varying current densities. (d) Pictures of scaling-up pouch cells with a cathode area size of ∼2.5 × 2.5 cm^2^ using the PAM organohydrogel electrolyte. (e) Cyclic stability performance of the HGe-LIG//Zn ZHC pouch cell at a current density of 1.35 A g^–1^.

To investigate the influence of DMSO on the PAM hydrogel electrolyte at the molecular level, Fourier transform infrared spectroscopy (FTIR) measurements were carried out. The FTIR spectra in Figure S23 (Supporting Information) show a blue shift of the O–H stretching vibration (2800–3800 cm^–1^) due to the addition of DMSO in PAM hydrogel. The result implies that DMSO perturbs the hydrogen-bonding network among H_2_O molecules.^59–61^ A hydrogen-bonding network was formed between DMSO and H_2_O, which weakens the interaction between Zn^2+^ and H_2_O and enhances the interaction between Zn^2+^ and DMSO. The DMSO decreases the hydrogen-bonding network between H_2_O molecules, strengthens the H–O covalent bonding, and changes the Zn^2+^ solvation structure (schematic representation in Figure S24, Supporting Information). This greatly stabilizes Zn^2+^ by significantly reducing the coordination between water and Zn^2+^ ions. These factors contribute to the high capacitance and long cycling stability observed in ZHCs using the PAM organohydrogel electrolyte.

## Conclusions

In summary, HGe and MGe were used in ZHCs, and we compared the EC performance of HGe-LIG and MGe-LIG heterostructures in aqueous, hydrogel, and organohydrogel electrolytes. The laser energy produces enough thermal and mechanical stress to break the micrometer-sized 2D HGe/MGe sheet into nanodimensions, which are uniformly distributed over the conductive LIG support. This unique dense structure enables effective ion access without causing an ion-sieving effect. A succession of ex situ characterization discloses that the charge storage process for both HGe-LIG and MGe-LIG heterostructures involves respective mechanisms, including the reversible chemical adsorption/desorption of Zn_4_SO_4_(OH)_6_.5H_2_O salt, intercalation of Zn^2+^ ions, etc. In combination with DFT calculations, we understand the adsorption preferences of a Zn atom in three highly symmetric sites of germanene, i.e., top, bridge, and hollow sites. Benefiting from high electrical conductivity, the HGe-LIG cathode delivers high specific capacity in aqueous (∼104 F g^–1^ @ 0.25 A g^–1^) and organohydrogel (∼87 F g^–1^ @ 0.25 A g^–1^) electrolytes, respectively. Whereas the more stable MGe-LIG cathode represents superior long-term stability (capacity retention 83%) compared to HGe-LIG (capacity retention 73%) after ∼12 k cycles in an aqueous electrolyte. Overall, the ability of the heterostructure to accommodate strain during long cycles, the short diffusion barrier of Zn^2+^ ions, the large electrolyte-accessible surface area enabled by the nanodimension morphology of HGe/MGe, and the conductive ion transport pathways due to LIG support the high EC performances in our study. Despite the impressive Zn^2+^ charge storage capacity, Ge is considerably more expensive compared to conventional cathode materials such as Mn-, V-, or C-based compounds.^62^ Ge also has a relatively high density (∼5.35 g cm^–3^) which has a negative impact on gravimetric and volumetric energy/power density as compared to lighter battery materials (like Li, C, Si, Sn, Al, Mg), thereby limiting its suitability for large-scale commercial applications.^63^ The present work partially addresses these issues by keeping the Ge content per electrode relatively low, minimizing both cost- and density-related drawbacks. This work shows that preparing 2D functionalized germanane heterostructures is a good prospect for ZHCs and has the potential to expand by numerous alkyl chains for alkali and transition metal (Na, K, Mg, Ca, Zn, and Al) battery applications.

## Materials and Methods

### Materials

Zn foil, zinc sulfate, polyacrylamide, *N*,*N*-methylenebis­(acrylamide) (MBAA, cross-linking agent), and potassium persulfate were purchased from Merck, Germany. Polyimide films (0.005″) were obtained from Fiedler Scientific Instruments, Czech Republic. HGe and MGe were also obtained from Merck, Germany.

### Synthesis of HGe and MGe

HGe and MGe were synthesized by following the protocols reported previously.^13,34^ In brief, CaGe_2_ crystals were added to cold 35% HCl and stirred gently for the next 10 days at −40 °C. The resulting dark brown-gray colored HGe powder was collected by filtration with cold 1 M HCl and deionized water.

For MGe synthesis, 0.4 g of CaGe_2_ crystals was first added to the CH_3_I, after which water and acetonitrile were added sequentially. The mixture was stirred slowly for 7 days, and the final MGe powder was collected by washing thoroughly with water and acetonitrile.

### Preparation of 2D HGe/MGe-LIG Heterostructures

A diode-pumped solid-state Nd:YAG laser (Oxford Lasers A-Series) operating at a wavelength of 532 nm was used to fabricate the structure. First, the uniform dispersion of MGe/HGe (1 mg/mL) was spin-coated on the precleaned polyimide films. The HGe/MGe-LIG heterostructure was then created using a single-step laser writing using a defocused lasing method. The laser parameters are tabulated in Table 1.

**Table 1:** Laser Parameters Used to Fabricate the Cathode

power	3 W
scanning speed	50 mm/s
pulse frequency	7 kHz
resolution	0.5 μm
central wavelength	532 nm

### Material Characterization

SEM imaging was conducted on an FEI VERIOS 460L, while elemental mapping was carried out by using a Tescan Mira 3 XMU equipped with an Oxford Instruments X-MAX EDS detector. Surface chemical composition was analyzed through XPS using a Kratos Analytical Axis Supra instrument with a monochromatic Al Kα source (1486.7 eV). The XPS spectra were calibrated to the C 1s peak at 284.8 eV and processed with CasaXPS software. Raman spectroscopy was performed across the 200–3000 cm^–1^ range using a Witec Alpha 300R spectrometer with a 532 nm laser. The surface roughness and height profiles were obtained by using a confocal laser scanning microscope (CLSM), specifically the Olympus Lext OLS4100 with a 405 nm laser. FTIR spectra were captured using a Vacuum FTIR Vertex70v spectrometer. The low-voltage electron microscope images were captured by using the LVEM 25E.

### Synthesis of PAM Organohydrogel and Hydrogel Electrolytes

The PAM organohydrogel electrolyte was synthesized as follows: 4 g of acrylamide, 4 mg of *N*,*N*-methylenebis­(acrylamide), and 10 mg of K_2_S_2_O_8_ were dissolved in a mixture of water and DMSO (5 mL of DI water and 5 mL of DMSO) and stirred magnetically at 700 rpm. The resulting solution was transferred to a glass Petri dish, which was then sealed with tape and heated in an oven at 70 °C for 24 h. The prepared PAM organohydrogel was subsequently soaked in a 2 M ZnSO_4_ electrolyte solution for 24 h prior to use.

The PAM hydrogel electrolyte was synthesized following the same procedure as the organohydrogel electrolyte but without adding DMSO.

### Electrochemical Measurements

The ZHCs were assembled using a Swagelok cell setup with He/MGe-LIG as the cathode, 2 M ZnSO_4_ or hydrogel/organohydrogel as the electrolyte, a glass fiber separator (Whatman GF/B), and zinc metal foil (0.1 mm thickness, 99.95% purity, Sigma-Aldrich) as the anode. Once assembled, the cells were sealed under ambient conditions and rested for 12 h to stabilize the open-circuit potential (OCP) before EC testing.

### Computational Details

Spin-polarized density functional theory (DFT) calculations were performed with the Perdew, Burke, and Ernzerhof (PBE) exchange and correlation functional^64^ and projected augmented wave (PAW) potentials^65,66^ as implemented in the Vienna Ab initio Simulation Package (VASP).^67^ The Grimme D3 empirical dispersion^68^ was used to introduce dispersion contributions. The wave functions were expanded in the plane-wave basis set with a minimum cutoff of 500 eV. To model the systems as extended periodic structures, periodic boundary conditions (PBC) were applied. The geometry optimizations were performed using the 3 × 3 × 1 Γ-centered Monkhorst–Pack *k*-point mesh.^69^ All the optimized structures were converged to forces of less than 10^–2^ eV/Å, and an electronic energy convergence criterion of 10^–6^ eV for each self-consistent cycle. The tetrahedron method with the Blöchl corrections^70^ was used for the density of states (DOS) calculations with a 9 × 9 × 1 Γ-centered Monkhorst–Pack *k*-point mesh. The strength of the interaction was evaluated by adsorption energies as *E*_ads_ = (*E*_system+Zn_ – *E*_system_ – *E*_Zn_), where *E*_system+Zn_, *E*_system_, and *E*_Zn_, denoted total energies of whole Zn@HGe­(/graphene) or Zn@MGe­(/graphene), HGe­(/graphene) or MGe­(/graphene), and Zn atom, respectively.

## Supplementary Material



## Notes

SEM, TEM images, EDX spectra, FTIR spectra, and XPS survey scan spectra of HGe/MGe powder and HGe-LIG/MGe-LIG films (Figures S1–S5, S7–S8); TGA spectra of LIG films (Figure S5); CV curve with capacitive and diffusive contribution, GCD profile, and cycle-resolved EIS spectra of HGe-LIG/MGe-LIG cathodes in aqueous electrolyte (Figures S9–S12, S16); ex situ XPS and in situ operando Raman spectra of HGe-LIG/MGe-LIG cathodes at various states of the discharge/charge process (Figures S13–S15); additional theoretical simulation results (Figure S17); GCD profile of HGe-LIG/MGe-LIG cathodes (Figure S18) and rate performances of HGe-LIG cathodes (Figure S19) in hydrogel electrolyte; GCD profile and corresponding series connection of the HGe-LIG//Zn ZHC pouch cell in organohydrogel electrolyte (Figures S21–S22); FTIR spectra of hydrogels (Figure S23); snapshot of transparent organohydrogel electrolyte (Figure S20); schematic of the hydrogen bond network within gel electrolytes (Figure S24); Raman, XPS, and EIS fitting results (Tables S1–S3); ZHC device performance with the current state-of-the-art (Table S4) (PDF)
