# Supplementary material for: Laser-Processed 2D Germanane on Graphene for Organohydrogel-Based Zinc-Ion Hybrid Capacitors
Source: ACS Nano. 2026 Feb 27;20(10):8275–88. doi: 10.1021/acsnano.5c13803 (PMC13001108; doi:10.1021/acsnano.5c13803)
Supplement: Supplementary file 1 [file nn5c13803_si_001.pdf]

Laser-Processed 2D Germanane on Graphene for Organohydrogel-Based Zinc-Ion Hybrid Capacitor

Sujit Deshmukh,[1] Keval K. Sonigara,[1] Rostislav Langer[2], Michal Otyepka[2,3], Martin Pumera*[1,4,5,6]

[1] *Future Energy and Innovation Laboratory, Central European Institute of Technology, Brno University of Technology, Purkyňova 123, 61200 Brno, Czech Republic.*

[2] *IT4Innovations, VSB – Technical University of Ostrava, 17. listopadu 2172/15, 708 00 Ostrava-Poruba, Czech Republic*

[3] *Regional Centre of Advanced Technologies and Materials, Czech Advanced Technology and Research Institute (CATRIN), Palacký University Olomouc, Olomouc, Czech Republic.*

[4] *Faculty of Electrical Engineering and Computer Science, VSB - Technical University of Ostrava, 17. listopadu 2172/15, 70800 Ostrava, Czech Republic*

[5] *Department of Medical Research, China Medical University Hospital, China Medical University, No. 91 Hsueh-Shih Road, Taichung, Taiwan.*

[6] *Department of Chemical and Biomolecular Engineering, Yonsei University, 50 Yonsei-ro, Seodaemun-gu, Seoul 03722, Korea*

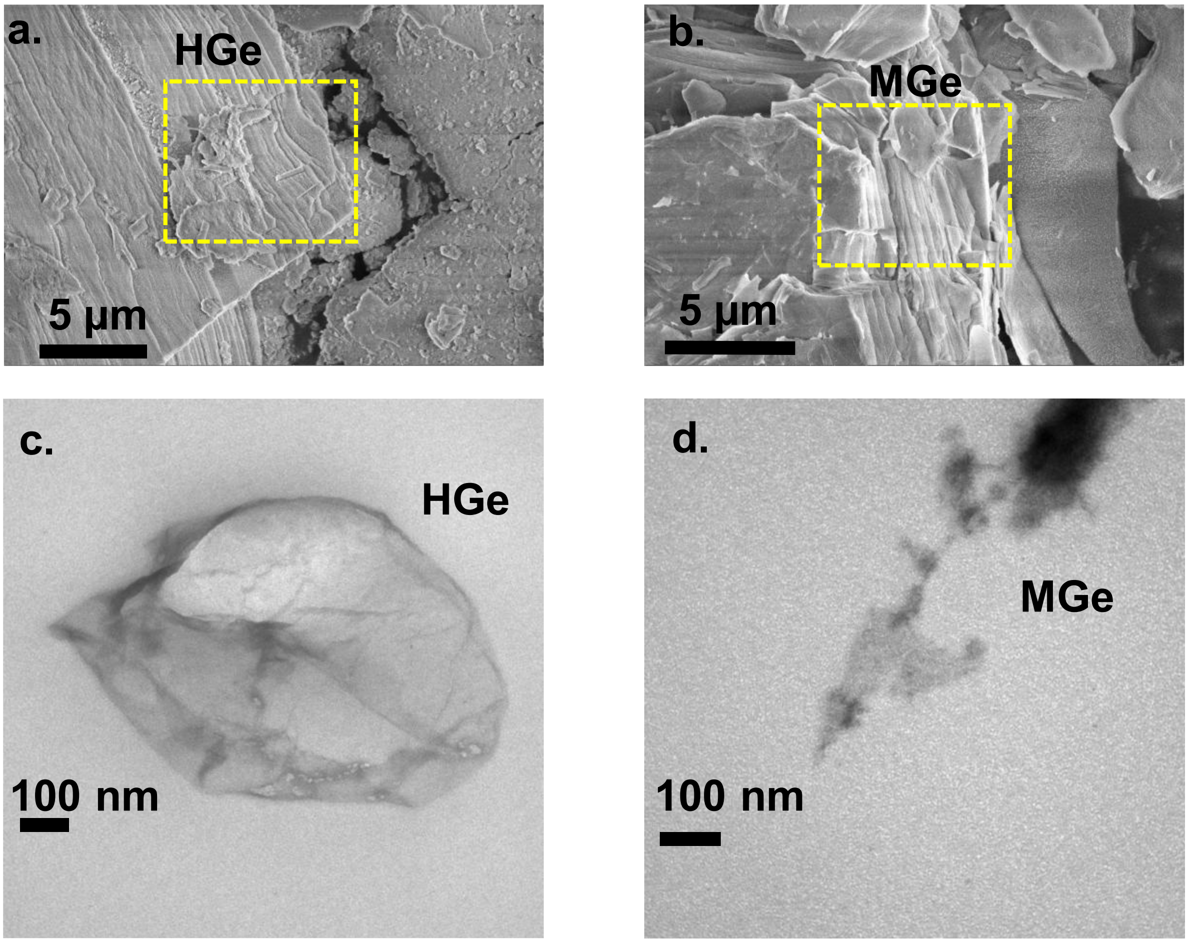

Figure S1. SEM top view of **(a)** HGe and **(b)** MGe powder. Typical TEM image of **(c)** HGe and **(d)** MGe sheet.

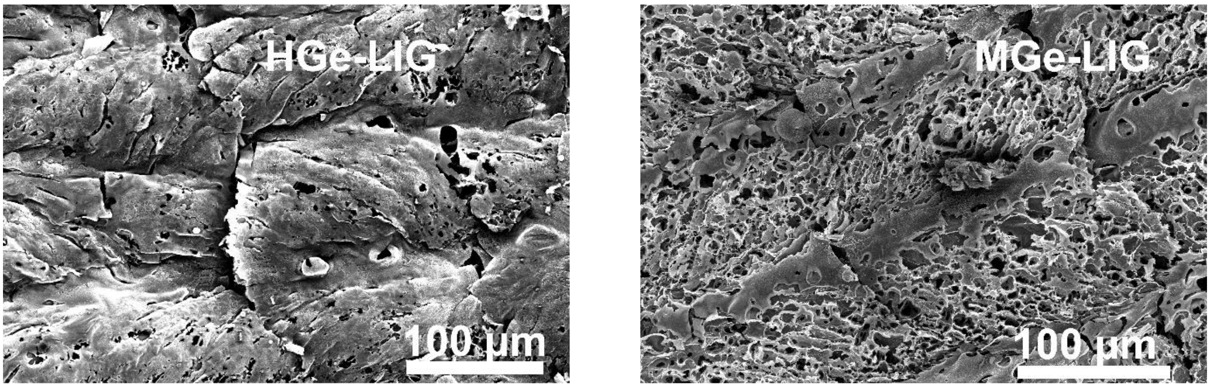

Figure S2. Low magnification SEM top view of HGe-LIG and MGe-LIG film.

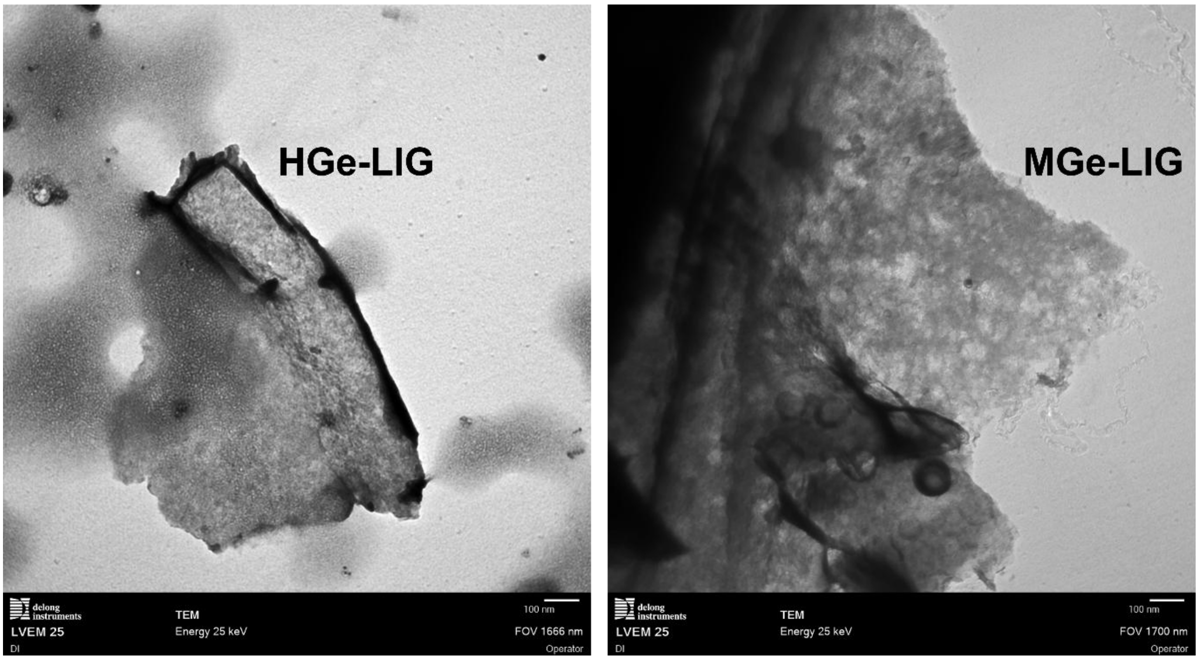

Figure S3. Low voltage TEM images of HGe-LIG and MGe-LIG film.

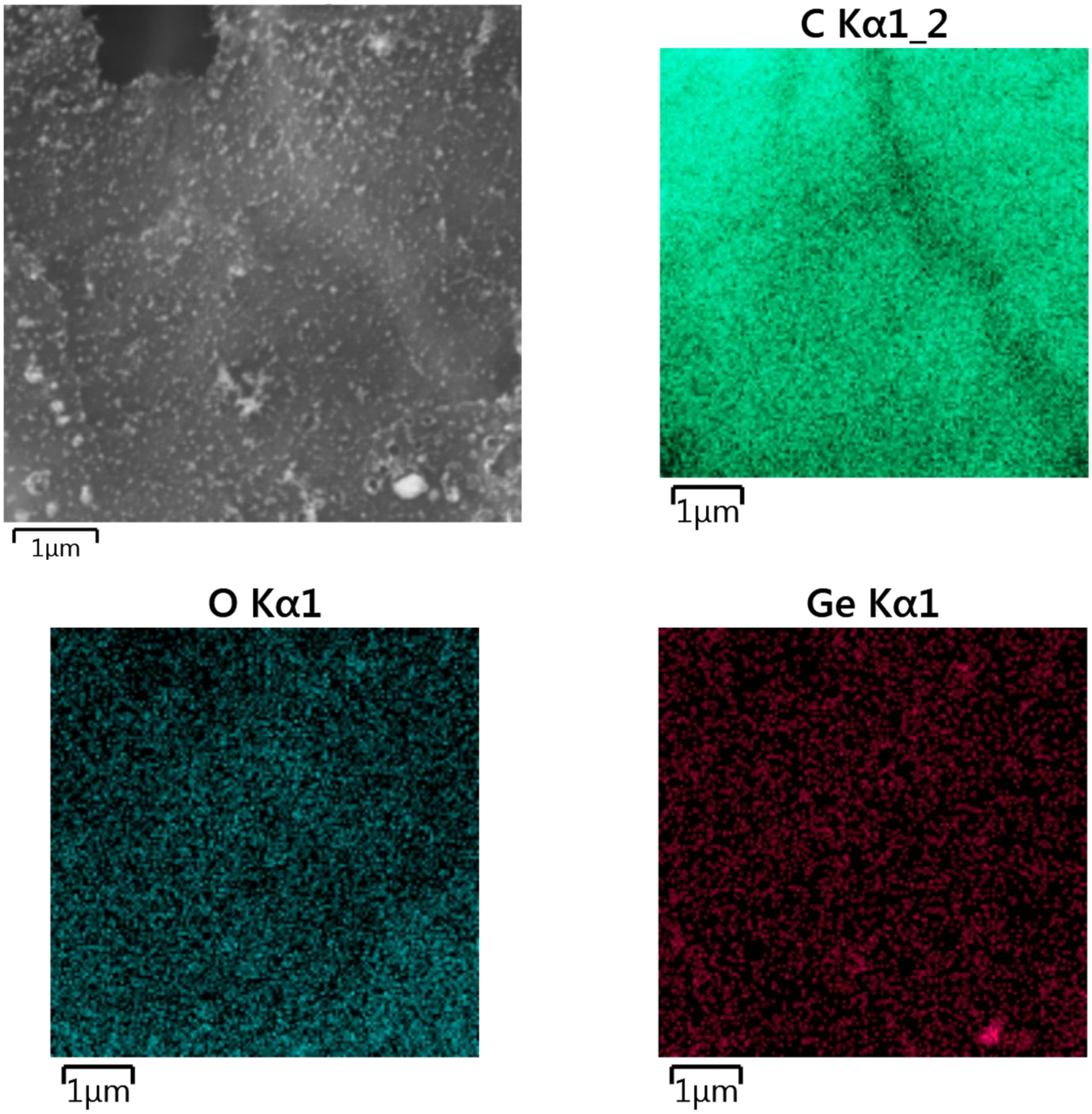

Figure S4. Energy-dispersive X-ray spectroscopy elemental mapping of HGe-LIG film showing the distribution of C, O, and Ge.

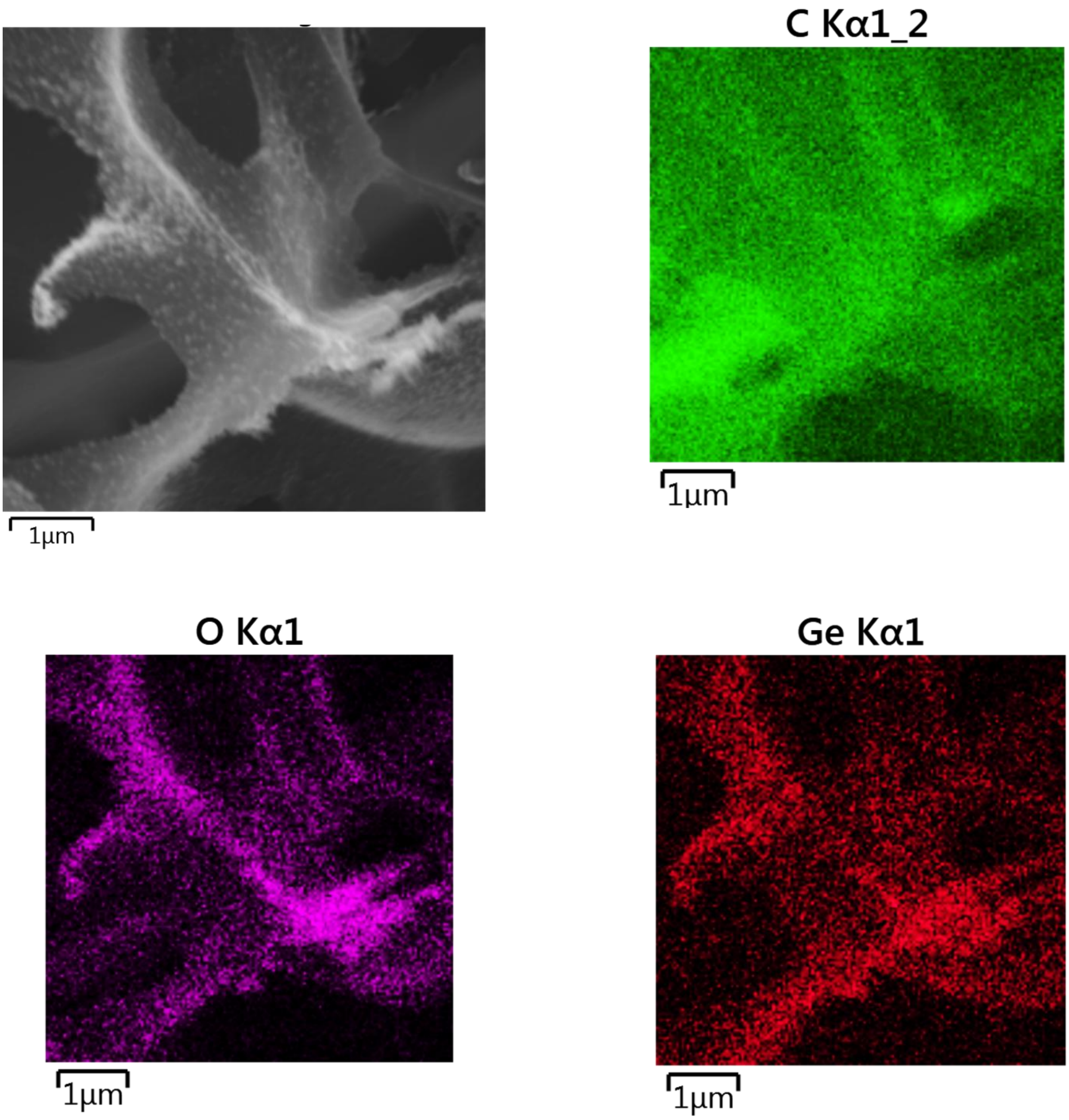

Figure S5. Energy-dispersive X-ray spectroscopy elemental mapping of MGe-LIG film showing the distribution of C, O, and Ge.

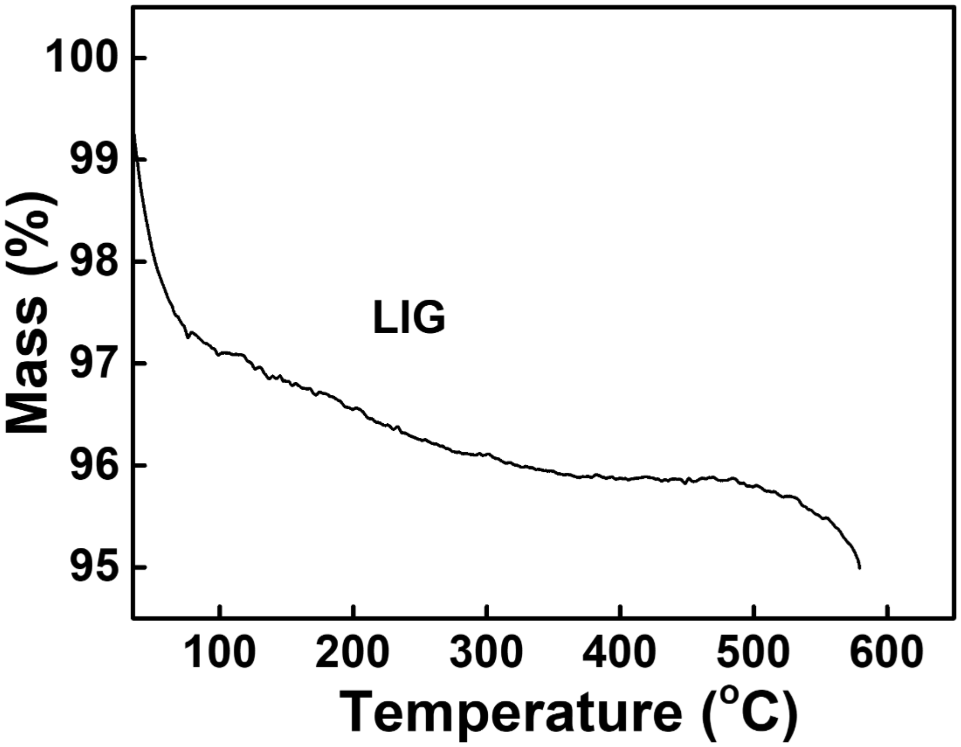

Figure S6. TGA spectra of LIG film.

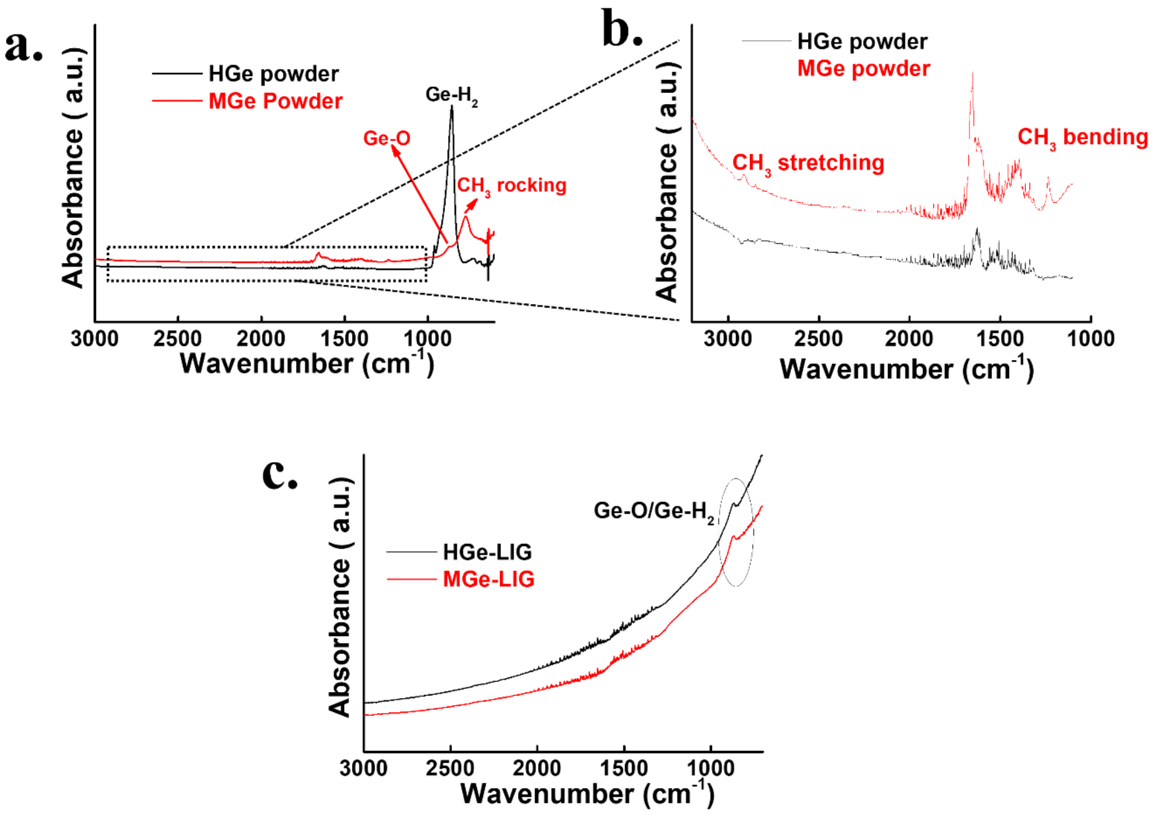

Figure S7. (a) FTIR spectra of HGe and MGe powder. **(b)** Zoomed view of the region (1000-3500 cm^{-1}) marked in dotted line in Figure a. **(c)** FTIR spectra of HGe-LIG and MGe-LIG film.

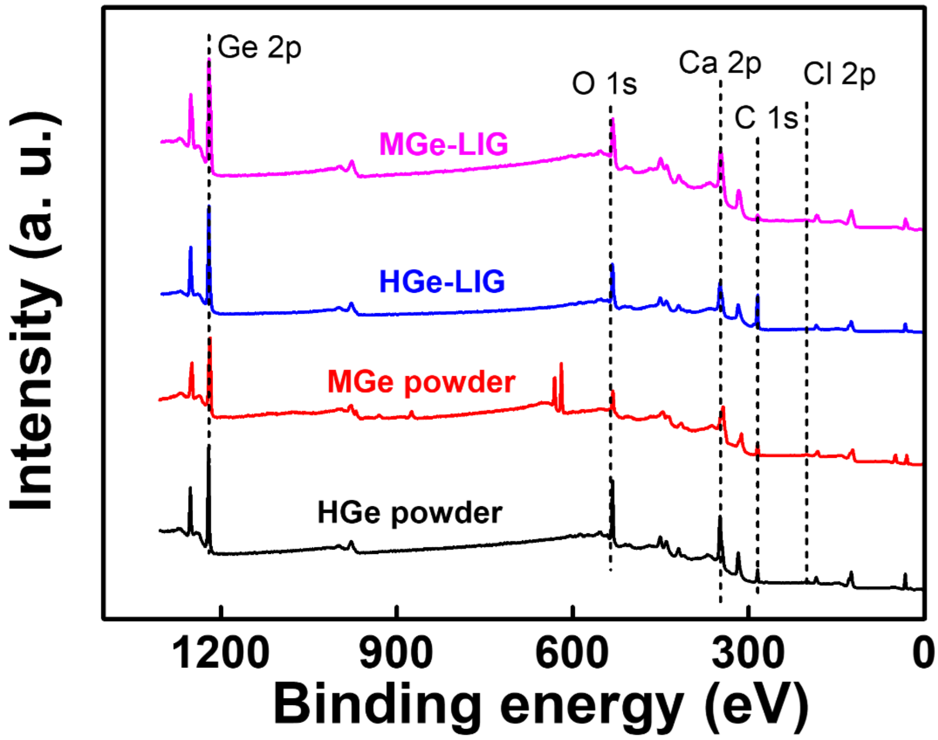

Figure S8. XPS survey scan spectra of HGe/MGe powder and HGe-LIG/MGe-LIG film.

Table S1. Extracted results from XPS survey spectra.

Sample	C (at%)	O (at%)	Ca (at%)	Cl (at%)	Ge (at%)
HGe powder	10.96	18.95	12.74	0.72	56.63
MGe powder	13.64	13.09	12.99	1.11	59.17
HGe-LIG	31.43	14.26	9.12	0.31	44.88
MGe-LIG	6.06	16.96	13.77	1.49	61.73

Table S2. XPS analysis of Ge 2p region before and after cycling performance.

**Sample**	**Elemental Ge (at%)**	**Ge-H/Ge-C (at%)**	**GeO/GeO_2 (at%)**
HGe powder	6.9	93.10	-
MGe powder	59.55	18.54	21.91
HGe-LIG	8.71	91.29	-
MGe-LIG	26.03	39.89	34.02
$HGe\text{-}LIG_{10000}$	-	33.84	66.16
$MGe\text{-}LIG_{10000}$	-	51.03	48.97

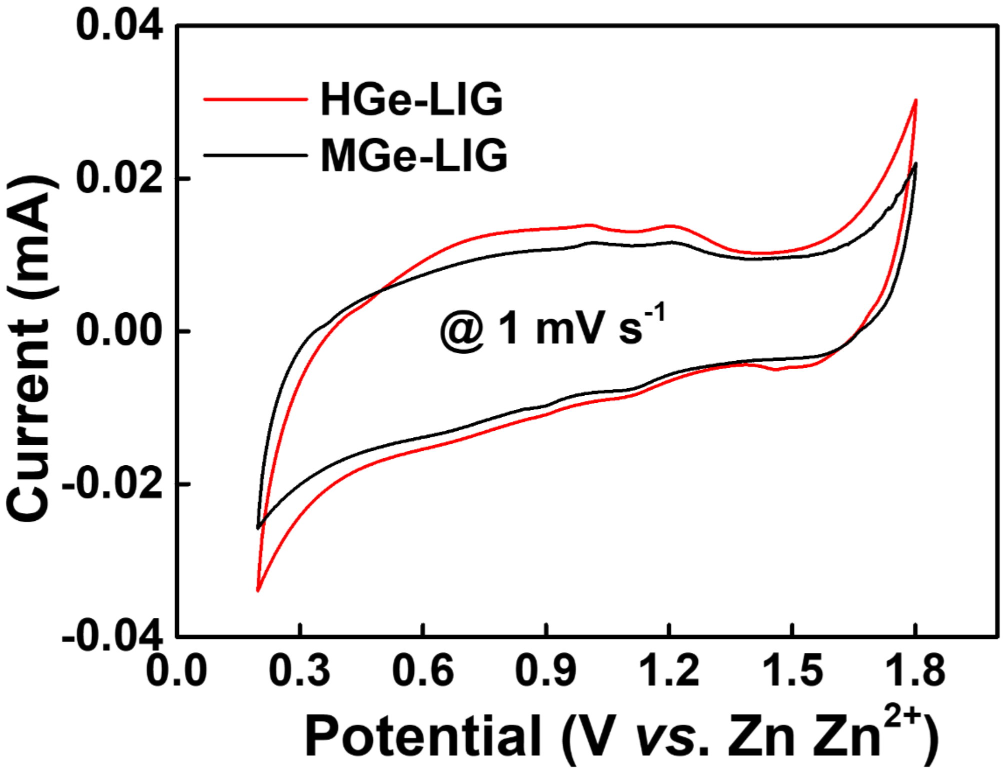

Figure S9. Comparative cyclic voltammetry curve of HGe-LIG and MGe-LIG cathode in 2 M $ZnSO_4$ electrolyte.

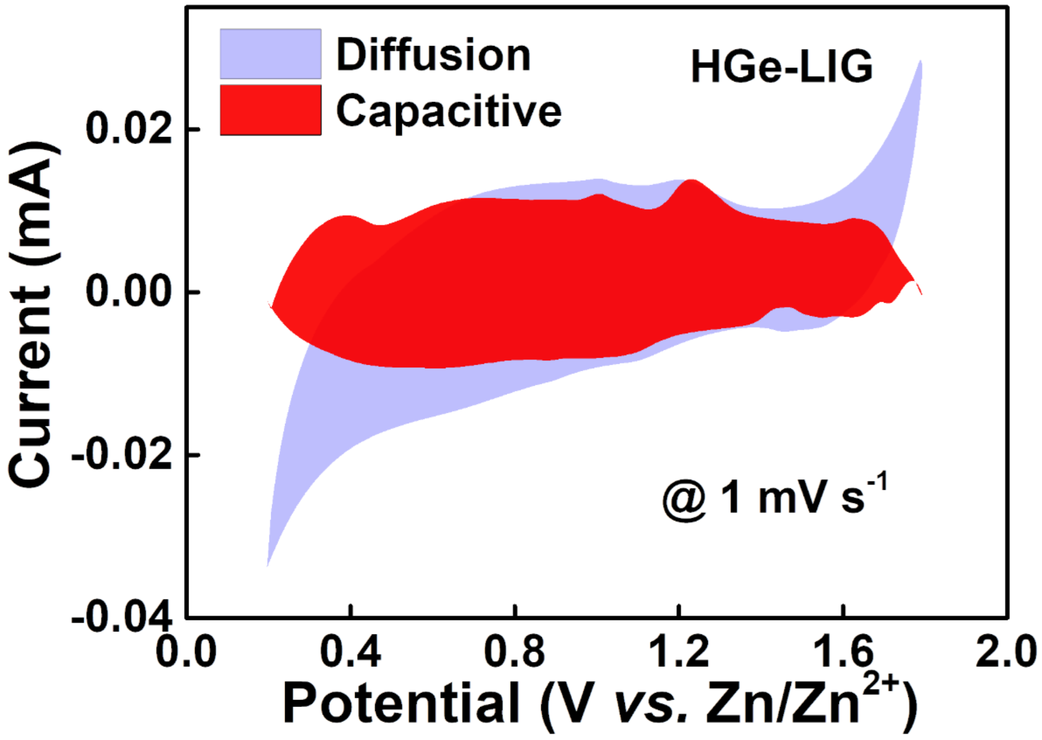

Figure S10. CV profile of HGe-LIG at 1 mV s^{-1} showing the capacitive (red region) and diffusive contribution (blue region). Electrolyte used 2 M $ZnSO_4$.

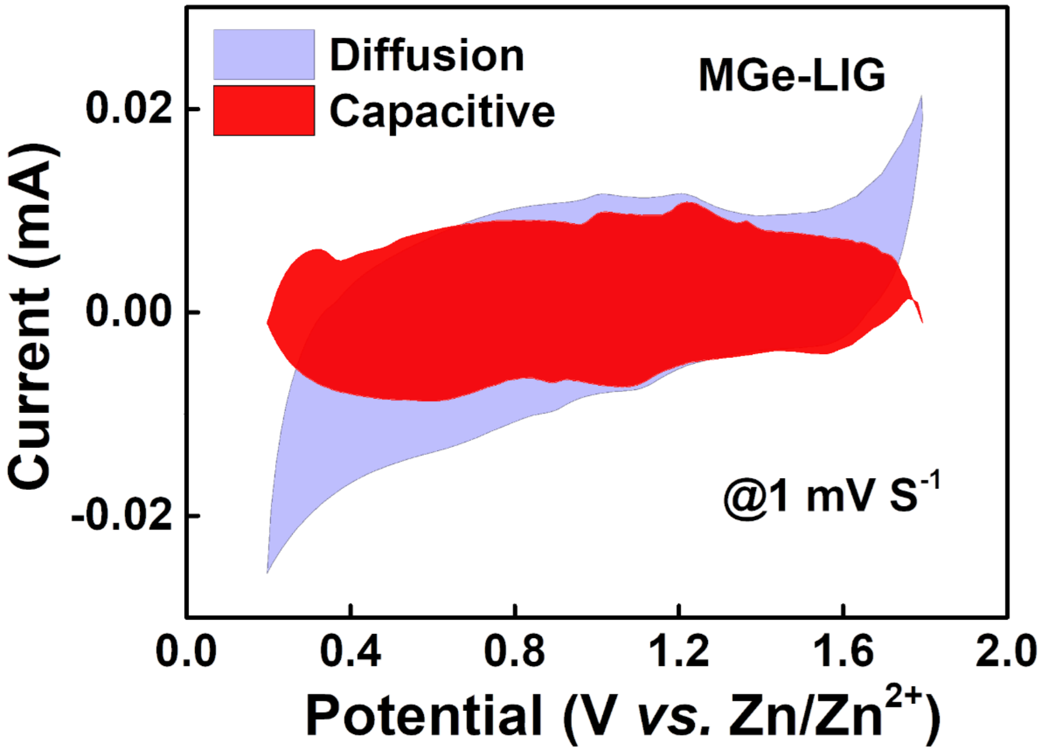

Figure S11. CV profile of MGe-LIG at 1 mV s^{-1} showing the capacitive (red region) and diffusive contribution (blue region). Electrolyte used 2 M $ZnSO_4$.

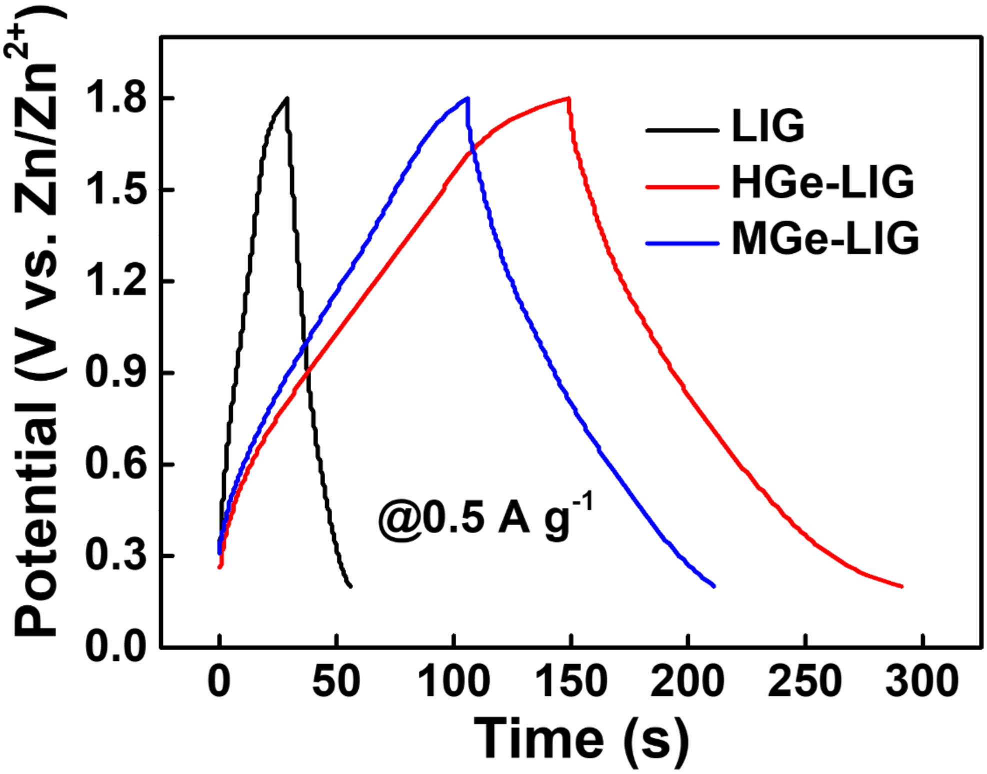

Figure S12. Comparison of GCD profiles for pristine LIG, HGe-LIG and MGe-LIG cathodes in 2 M $ZnSO_4$ electrolyte.

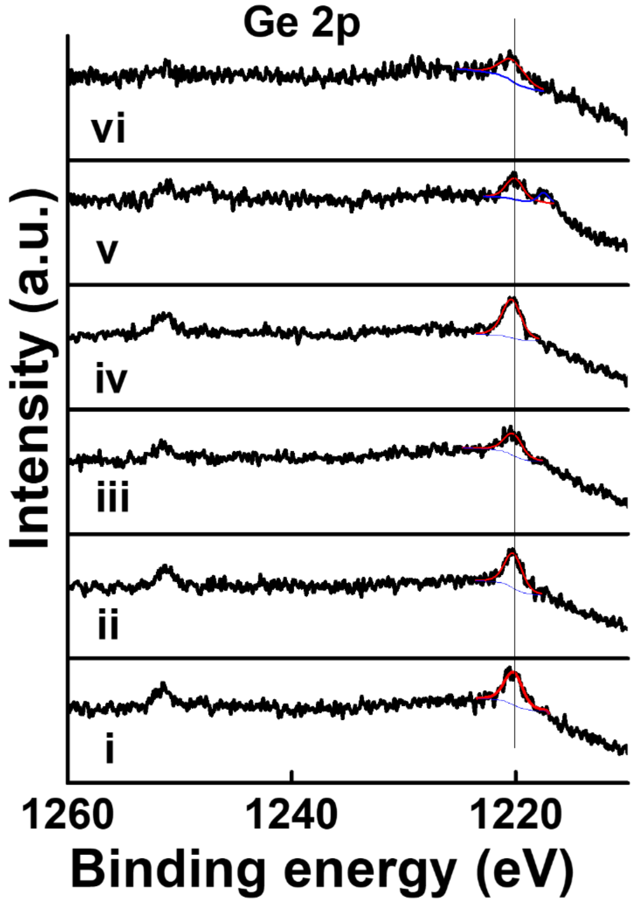

Figure S13. Ge 2p high-resolution ex-situ XPS spectra of HGe-LIG cathode.

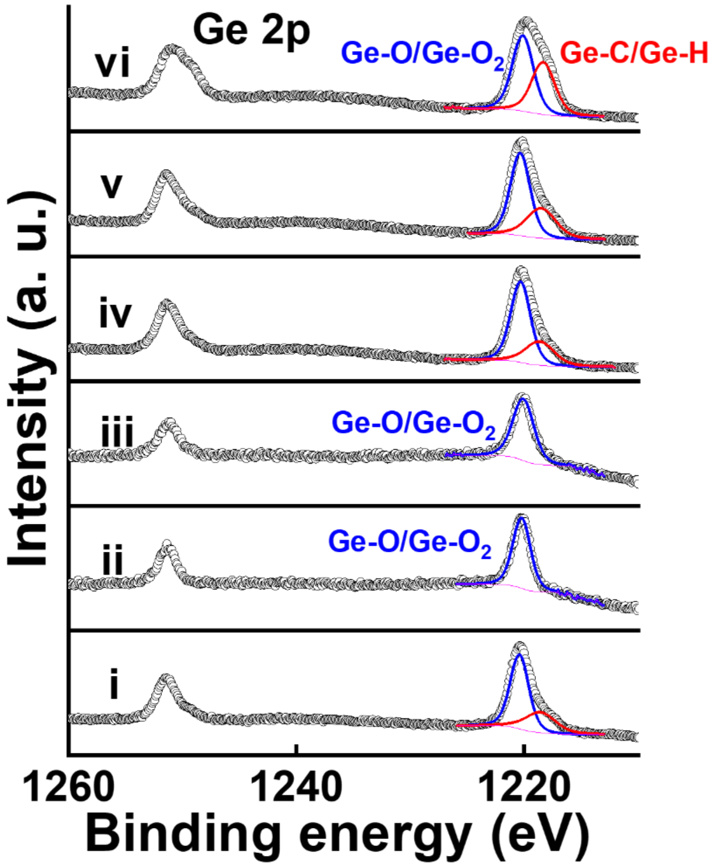

Figure S14. Ge 2p high-resolution ex-situ XPS spectra of MGe-LIG cathode.

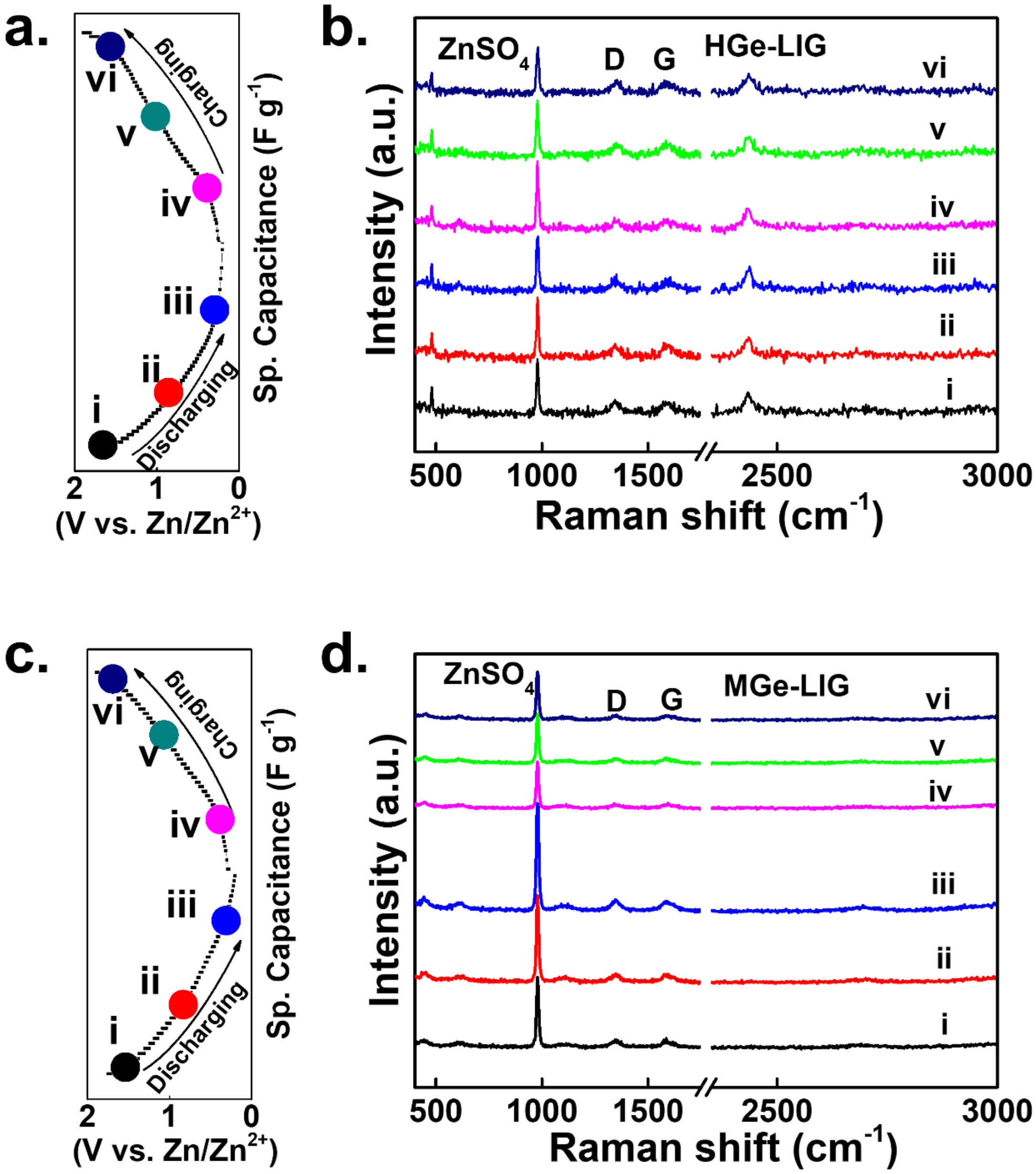

Figure S15. (a, c) Discharge/charge profile of HGe-LIG, MGe-LIG cathode. **(b, d)** In-situ operando Raman spectra of HGe-LIG and MGe-LIG cathode at various states of discharge/charge process.

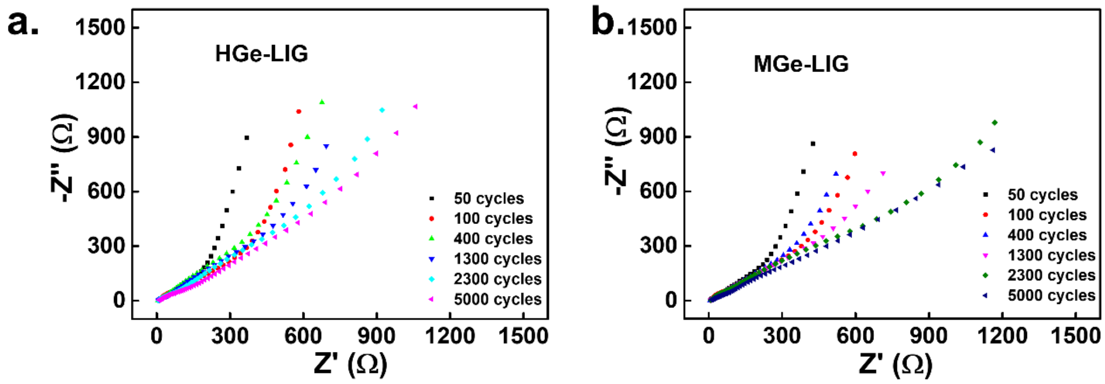

Figure S16. Cycle resolved EIS spectra of (a) HGe-LIG and **(b)** MGe-LIG cathode in 2 M $ZnSO_4$ electrolyte.

Table S3. The change in the charge transfer resistance (R_{ct}) of HGe-LIG and MGe-LIG cathode with varying charge-discharge cycles.

Cycle number	Charge transfer resistance (R_{ct}) of HGe-LIG in Ω	Charge transfer resistance (R_{ct}) of MGe-LIG in Ω
50	25.7	68.3
100	42.5	158.6
400	43.2	167.1
1300	164.31	184.9
2300	195.2	192.7
5000	268.9	198.9

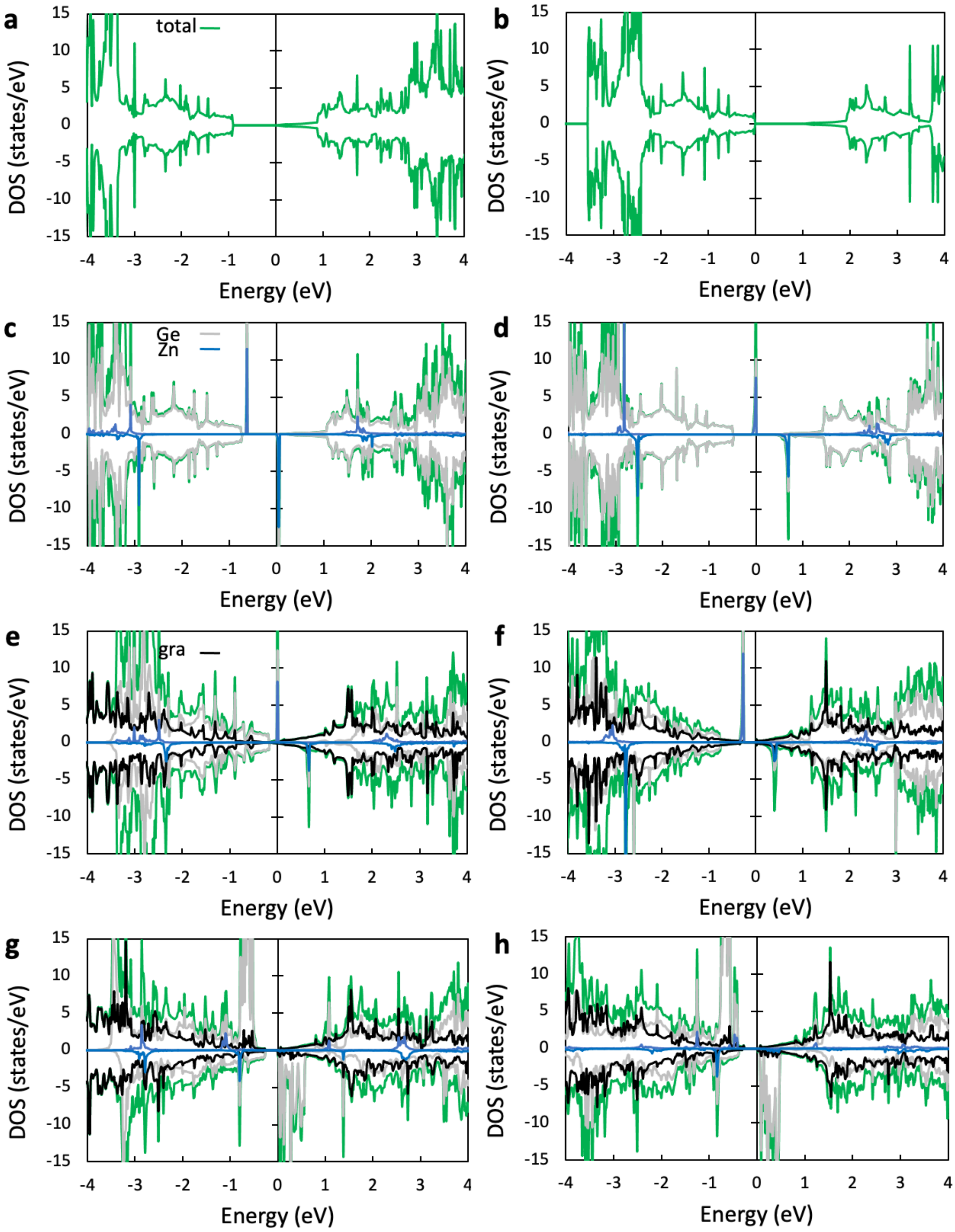

Figure S17. Atom-decomposed DOS of **(a)** MGe, **(b)** HGe, **(c)** Zn@MGe, **(d)** Zn@HGe, **(e)** Zn@MGe/graphene, **(f)** Zn@HGe/graphene, **(g)** Zn@MGe/graphene (single-side functionalization), and **(h)** Zn@HGe/graphene (single-side functionalization).

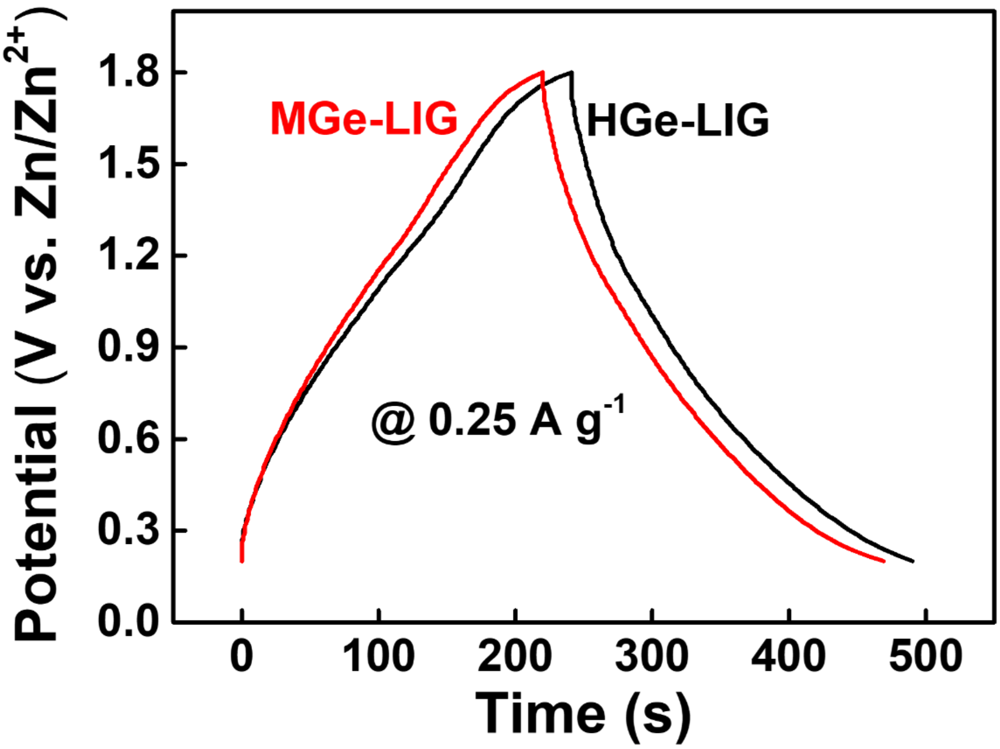

Figure S18. Comparison of GCD profiles for HGe-LIG and MGe-LIG cathodes at a current density of 0.25 A g^{-1} in hydrogel electrolyte.

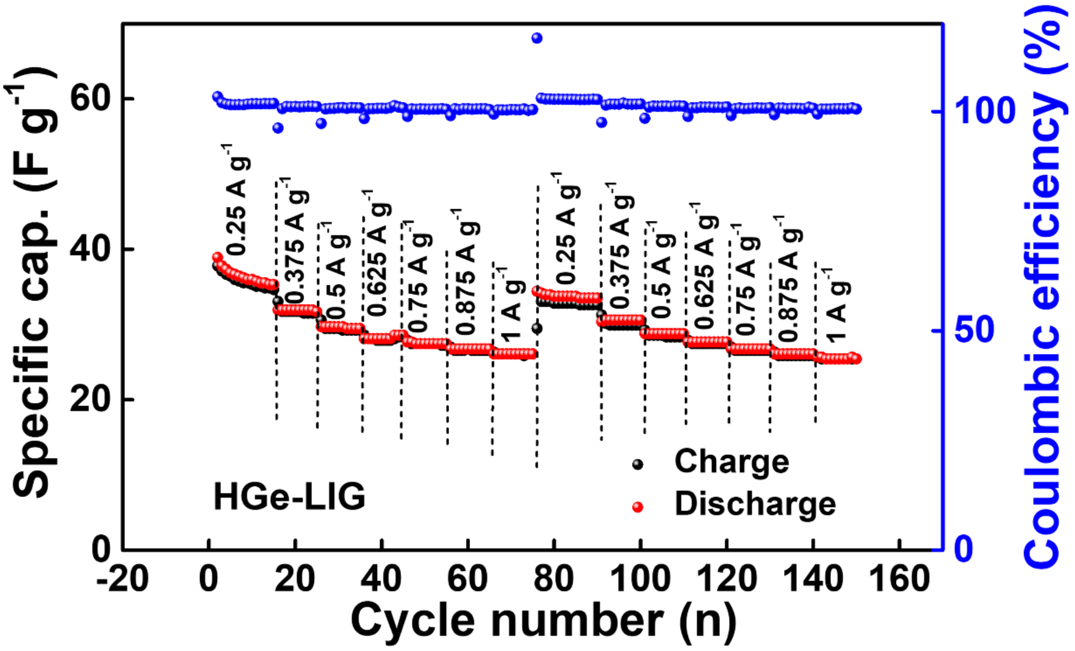

Figure S19. Rate performances of HGe-LIG cathode at varying current densities. The experiment was carried out in hydrogel electrolyte.

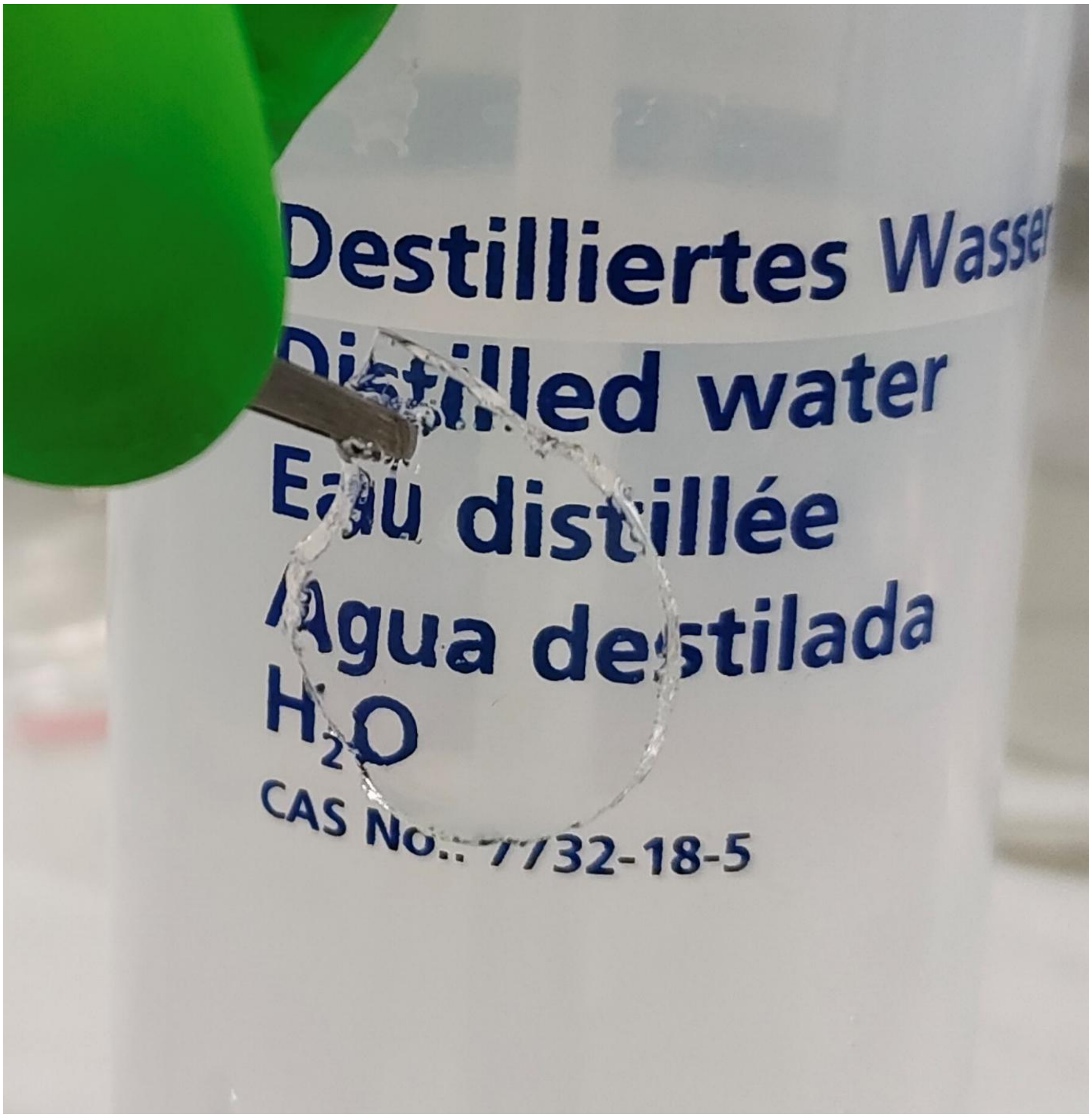

Figure S20. Snapshot of transparent organohydrogel electrolyte.

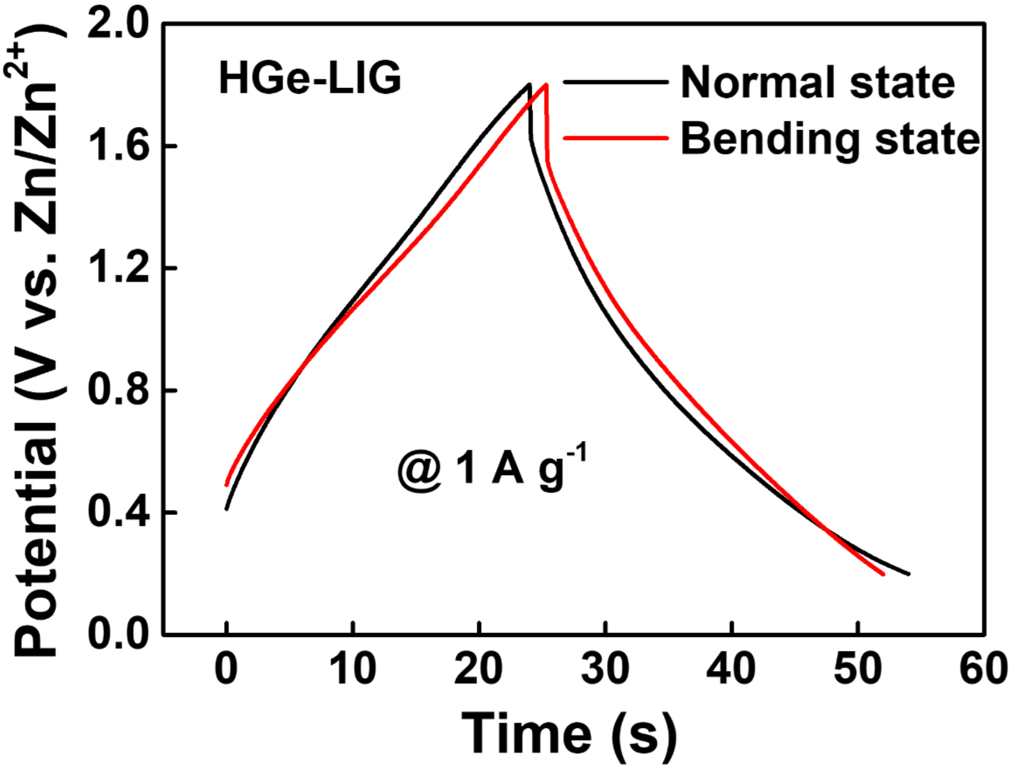

Figure S21. Charge-Discharge profiles of HGe-LIG//Zn ZHC pouch cell device under normal and bending states. The experiment was carried out in organohydrogel electrolyte.

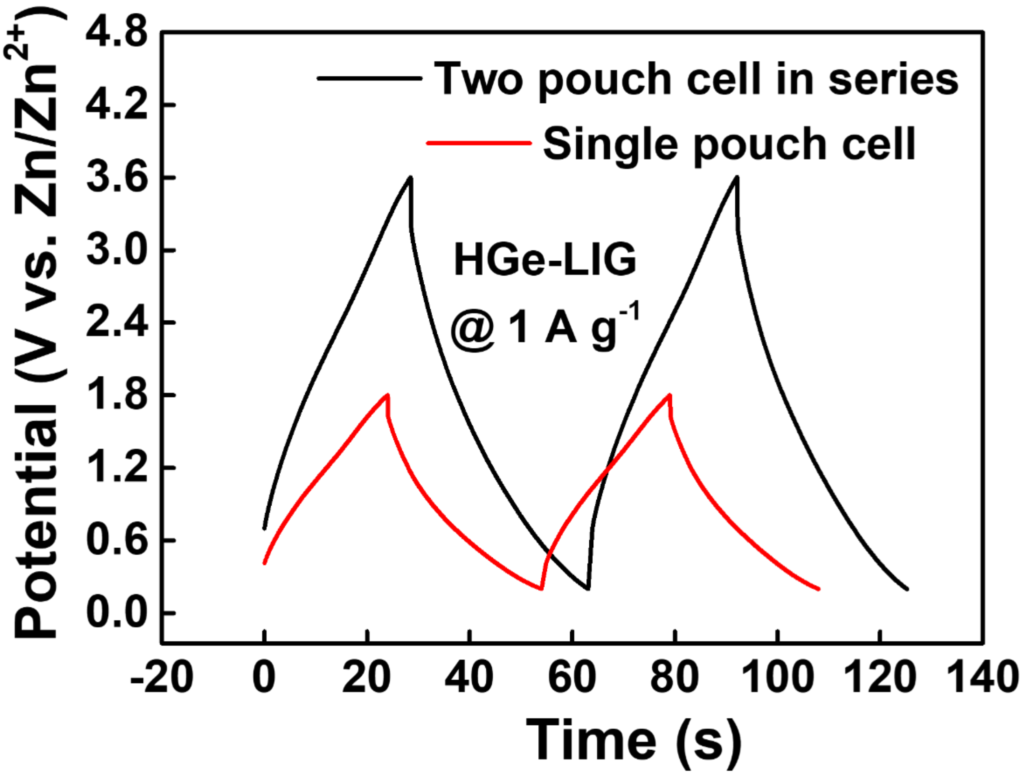

Figure S22. Charge-Discharge profiles of HGe-LIG//Zn ZHC pouch cell devices connected in series. The experiment was carried out in organohydrogel electrolyte.

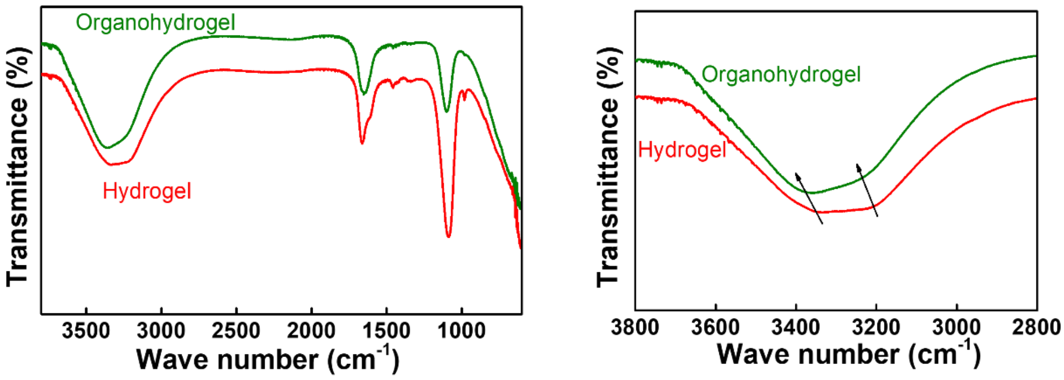

Figure S23. FTIR spectra of PAM hydrogel and PAM organohydrogel (with DMSO).

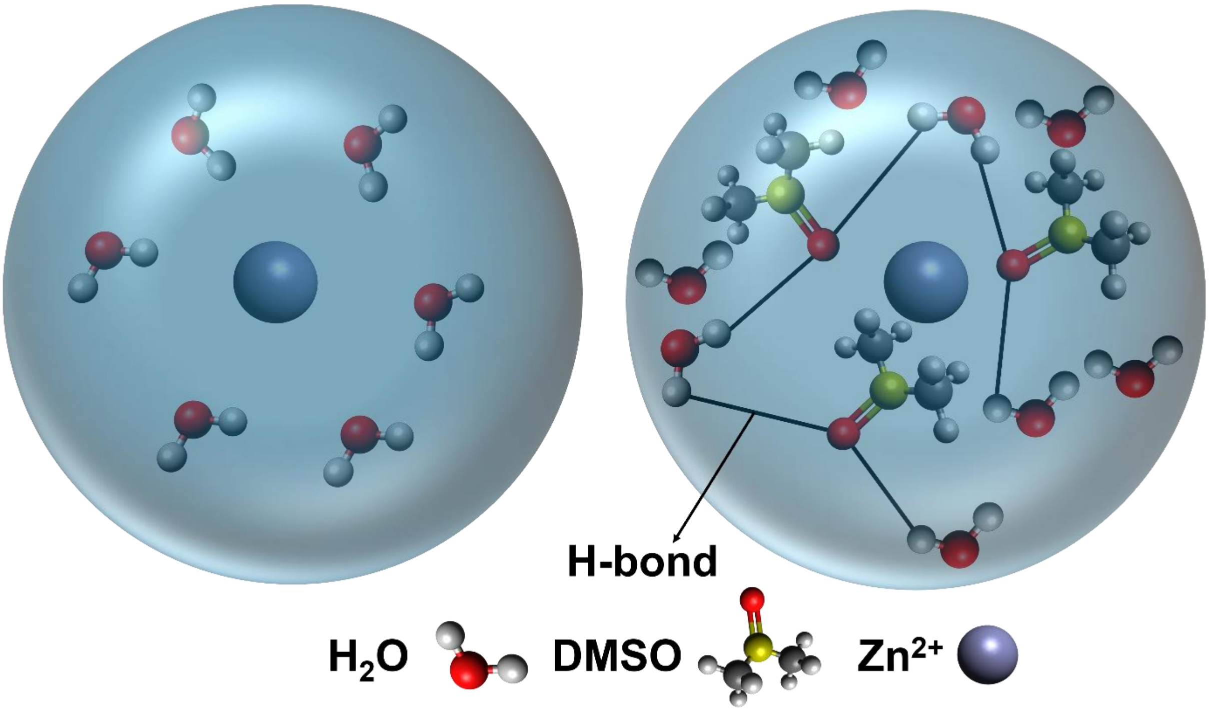

Figure S24. H-bond between DMSO and H_2O during the Zn^{2+} solvation. The blue circle represents the sheath structure of Zn^{2+}.

Table S4. Device performance comparison of HGe-LIG/MGe-LIG ZHC with other reported literature.

Electrode materials	Potential window	Electrolyte	Capacitance or capacity	Cycling stability	*Ref.*
HGe attached to LIG current collector<br><br>MGe attached on LIG current collector	0.2-1.8 V<br><br>0.2-1.8 V	2 M $ZnSO_4$<br><br>2 M $ZnSO_4$	104 F g^{-1} @ 0.25 A g^{-1}<br><br>46 F g^{-1} @ 0.25 A g^{-1}	73% after 12k cycles<br><br>83% after 12k cycles	Our Work
2D Silicene Nanosheets	0-1.8 V	21 M $LiN(SO_2CF_3)_2$ (LiTFSI) and 1 M $Zn(CF_3SO_3)_2$ into deionized (DI) water	~14.5 mF cm^{-2} @0.05 mA cm^{-2}	112% after 10k cycles	[1]
Few-Layer Siloxene	0-1.8 V	21 M LiTFSI (12.058 g) and 1 M $Zn(CF_3SO_3)_2$ (0.728 g) in 2 mL of deionized water	~6.7 mF cm^{-2} @0.05 mA cm^{-2}	94.3% after 10k cycles	[2]
N-doped mesoporous graphitic carbon (N-mgc) by utilizing polystyrene-*block*-poly(2-vinlypyridine) copolymer (PS-*b*-P2VP)	0-1.8 V	2 M $Zn(CF_3SO_3)_2$ aqueous solution	~43 F g^{-1} @0.2 A g^{-1}	80% after 10k cycles	[3]
$NiPS_3$/graphene	0.5-1.5 V	$ZnSO_4$/PVA	~88 F g^{-1} @0.2 A g^{-1}	86% after 1k cycles	[4]
p-phenylenediamine-intercalated MXene (PDA-MXene)	0.2-1.1 V	2 M $ZnSO_4$	~124 F g^{-1} @0.2 A g^{-1}	85% after 1k cycles	[5]
3D gold interdigitated electrodes (3D Au IDEs) as highly porous current collectors, loaded with zinc (Zn) as the anode and hybrid activated carbon	0.6-1.4 V	1 M $ZnSO_4$ gel	~1.3 µAh cm^{-2}@0.2 mA cm^{-2}	78% after 5k cycles	[6]

coated with PEDOT (ACPEDOT) as the cathode.					
High-mass loading three-dimensionally (3D) printed Graphene-carbon nanotube (Gr–C) cathode	0-1.8 V	2 M ZnSO4 and 2 M NaCl	~0.84 mAh cm^{-2} @3 mA cm^{-2}	~79% after 5k cycles	7
MXene/rGO Foam	0.6-1.4 V	Gelatin in 1 M ZnSO4 electrolyte	~83.96 mF cm^{-2} @0.5 mA cm^{-2}	~80.8% after 1.4k cycles	8
Dry laser-assisted fabrication of F-doped graphene electrodes	0.2-1.6 V	1 M $Zn(CH_3COO)_2$	~0.84 µAh cm^{-2} @1 mA cm^{-2}	~65% after 1.4k cycles	9
Laser-processed vertically aligned reduced graphene oxide/ tannic acid arrays	0.2-1.8 V	$ZnSO_4$/gelatin	~136.6 µAh cm^{-2} @0.2 mA cm^{-2}	~70% after 5k cycles	10
Fluorinated laser-induced graphene	0.2-1.8 V	PVA/$ZnCl_2$ gel	~42.32 mF cm^{-2} @0.1 mA cm^{-2}	~79.4% after 7k cycles	11
Laser-assisted fabrication of turbostratic graphene electrodes	0.2–1.6 V	1 M $Zn(CH_3COO)_2$ aqueous electrolyte	~20.7 µAh cm^{-2} @0.5 mA cm^{-2}	~74% after 10k cycles	12

References:

(1) Guo, Q.; Liu, J.; Bai, C.; Chen, N.; Qu, L. 2D Silicene Nanosheets for High-Performance Zinc-Ion Hybrid Capacitor Application. *ACS Nano* **2021**, *15* (10), 16533–16541. https://doi.org/10.1021/acsnano.1c06104.

(2) Guo, Q.; Han, Y.; Chen, N.; Qu, L. Few-Layer Siloxene as an Electrode for Superior High-Rate Zinc Ion Hybrid Capacitors. *ACS Energy Lett.* **2021**, *6* (5), 1786–1794. https://doi.org/10.1021/acsenergylett.1c00285.

(3) Kim, K.-W.; Park, B.; Kim, J.; Seok, H.; Kim, T.; Jo, C.; Kim, J. K. Block Copolymer-Directed Facile Synthesis of N-Doped Mesoporous Graphitic Carbon for Reliable, High-Performance Zn Ion Hybrid Supercapacitor. *ACS Appl. Mater. Interfaces* **2023**, *15* (50), 57905–57912. https://doi.org/10.1021/acsami.3c02791.

(4) Sonigara, K. K.; Vaghasiya, J. V; Mayorga-Martinez, C. C.; Pumera, M. Flexible Energy Storage Patch Based on NiPS3/Graphene Zinc-Ion Hybrid Supercapacitor for Integrated Biosensors. *Chem. Eng. J.* **2023**, *473*, 145204. https://doi.org/10.1016/j.cej.2023.145204.

(5) Peng, M.; Wang, L.; Li, L.; Tang, X.; Huang, B.; Hu, T.; Yuan, K.; Chen, Y.

Manipulating the Interlayer Spacing of 3D MXenes with Improved Stability and Zinc-Ion Storage Capability. *Adv. Funct. Mater.* **2022**, *32* (7), 2109524. https://doi.org/10.1002/adfm.202109524.

(6) Fan, Y.; Naresh, N.; Zhu, Y.; Wang, M.; Boruah, B. D. Design of Porous 3D Interdigitated Current Collectors and Hybrid Microcathodes for Zn-Ion Microcapacitors. *ACS Nano* **2025**, *19* (13), 13314–13324. https://doi.org/10.1021/acsnano.5c00917.

(7) Nagaraju, G.; Tagliaferri, S.; Panagiotopoulos, A.; Och, M.; Quintin-Baxendale, R.; Mattevi, C. Durable Zn-Ion Hybrid Capacitors Using 3D Printed Carbon Composites. *J. Mater. Chem. A* **2022**, *10* (29), 15665–15676. https://doi.org/10.1039/D2TA03488C.

(8) Zhang, H.; Wei, Z.; Wu, J.; Cheng, F.; Ma, Y.; Liu, W.; Cheng, Y.; Lin, Y.; Liu, N.; Gao, Y.; Yue, Y. Interlayer-Spacing-Regulated MXene/RGO Foam for Multi-Functional Zinc-Ion Microcapacitors. *Energy Storage Mater.* **2022**, *50*, 444–453. https://doi.org/10.1016/j.ensm.2022.05.033.

(9) Samartzis, N.; Bhorkar, K.; Sygellou, L.; Bellou, E.; Boukos, N.; Chrissanthopoulos, A.; Yannopoulos, S. N. Dry Laser-Assisted Fabrication of F-Doped Graphene Electrodes: Boosting Performance of Zn-Ion Hybrid Capacitors. *Chem. Eng. J.* **2025**, *507*, 160505. https://doi.org/10.1016/j.cej.2025.160505.

(10) Chen, Y.; Xiao, L.; Li, Y.; Qiu, J.; Zang, L.; Yang, C. Laser-Assisted Preparation of Vertically Aligned Reduced Graphene Oxide/Tannic Acid Arrays for Flexible Aqueous Zinc-Ion Hybrid Capacitors. *Appl. Surf. Sci.* **2024**, *665*, 160230. https://doi.org/10.1016/j.apsusc.2024.160230.

(11) Dong, M.; Mu, Y.; Zhou, L.; Zhao, Y.; Zhang, X.; Tan, D.; Pan, X.; Wei, H. Fluorinated Laser-Induced Graphene towards High Performance Zn-Ion Hybrid Supercapacitors. *J. Alloys Compd.* **2024**, *973*, 172846. https://doi.org/10.1016/j.jallcom.2023.172846.

(12) Samartzis, N.; Bhorkar, K.; Athanasiou, M.; Sygellou, L.; Dracopoulos, V.; Ioannides, T.; Yannopoulos, S. N. Direct Laser-Assisted Fabrication of Turbostratic Graphene Electrodes: Comparing Symmetric and Zinc-Ion Hybrid Supercapacitors. *Carbon N. Y.* **2023**, *201*, 941–951. https://doi.org/10.1016/j.carbon.2022.09.076.
